# Presence of Emerging Contaminants in UK Honey—Human Pharmaceuticals a Concern for Honeybees?

**DOI:** 10.1021/acs.jafc.5c10414

**Published:** 2026-03-06

**Authors:** John Nightingale, Ben A. Woodcock, Narmin Garazade, Richard F. Pywell, Laura J. Carter

**Affiliations:** † School of Geography, The University of Leeds, Leeds LS29JT, U.K.; ‡ UK Centre for Ecology & Hydrology, Crowmarsh Gifford, Wallingford OX10 8BB, U.K.; § water@leeds, The University of Leeds, Leeds LS29JT, U.K.

**Keywords:** honey, foraging, QuECHERS, high-resolution mass spectrometry (HR-MS), confidence, emerging contaminants, human pharmaceutical

## Abstract

Emerging contaminants can accumulate in water, soils, and crops; however, little is known about the potential exposure to honeybees. Using samples collected surrounding arable farming in Great Britain and nontarget techniques, we identified 119 suspect chemicals in hives. On average, each hive contained 6.8 (±3.01) active ingredients, these included human pharmaceuticals (64%), industrial chemicals (10%), surfactants (8%), and plasticizers (5%). Elevated concentrations of the anti-inflammatory flurandrenolide (582.3 ± 348.4 ng/g), the nonsteroidal anti-inflammatorydrug aspirin (358.2 ± 390.1 ng/g), the fungicide azoxystrobin (298.5 ± 159.9 ng/g), the antihypertensive methyldopa (123.4 ± 60 ng/g), and the anticonvulsant carbamazepine (79.97 ± 54.2 ng/g) were identified. Elevated concentrations of human-origin contaminants of emerging concern (CECs), and their increased frequency in arable areas, indicate that the reuse of contaminated fertilizers contributes to accumulation in hives across English landscapes. Critically, most of these contaminants lack toxicity data for honeybees, making it impossible to assess their acute or chronic risks.

## Graphical abstract

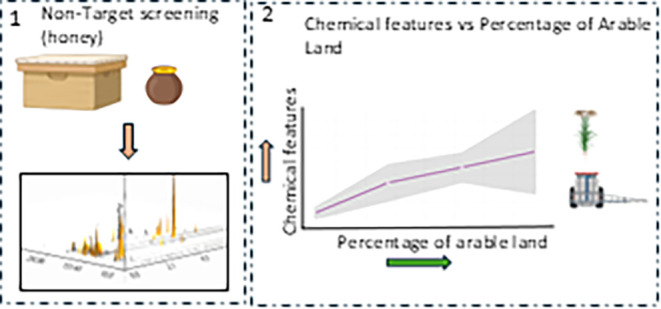

## 1 Introduction

Anthropogenic activity has directly resulted in the continuous release of contaminants into the environment that pose a potential risk to native biodiversity.^1^ Many of these are bioactive and exert negative effects on native and nontarget biota which may be aggravating not only the current biodiversity crisis but also potentially hindering critical ecosystem functioning.^2^ In terrestrial agricultural systems, select contaminants, like pesticides, are routinely considered within regulatory frameworks with their risks to nontarget taxa quantified to some extent.^3^ However, contaminants of emerging concern (CECs) comprise growing classes of chemicals that are present in the environment with potential risks toward biota. CECs comprise a broad range of chemicals including pharmaceuticals (human/veterinary), biocides, personal care products, surfactants, plasticizers, and per- and polyfluoroalkyl substances (PFAS). The hazards posed by such contaminants are widely reported in aquatic systems via the direct release of treated and untreated wastewater and urban drainage, yet remains poorly defined for terrestrial systems.^4,5^ Even so CECs are increasingly detected in soils, sediments, and aquatic systems following their release into the environment via a range of pathways.^6^ In agricultural systems, the application of treated sewage sludge (biosolids), wastewater treatment residues, wastewater, and animal manures or slurry has been linked to the widespread occurrence of CECs.^7–9^ These materials are reused on a significant scale. In the UK alone, approximately 96 million tonnes of animal manure, 3.6 million tonnes of biosolids/sludge, and 6.5 million tonnes of digestates are applied to land annually.^10–14^

Recent research has identified over 100 CECs in sewage sludge including pharmaceuticals, pesticides, flame retardants, and industrial chemicals.^15–17^ This includes recent quantification of CECs in treated sludges from UK wastewater treatment plants.^18^ Several of these compounds including benzodiazepines, anticonvulsants, antibiotics, analgesics, and nonsteroidal anti-inflammatory drugs (NSAIDs) are known to persist in soils, where they may cross root membranes and be taken up by plants, including agricultural crops.^19–21^ Although plant uptake of CECs can be limited by processes such as enzymatic metabolism, protein adsorption, and ion trapping,^22^ it is increasingly evident that compounds with specific physicochemical properties—particularly those with a log *p* < 3.5, molecular weight < 350 g/mol, and theoretical p*K*_a_ between 1 and 7—are more likely to translocate to above-ground plant tissues.^22–24^ For example, Carter et al.,^25^ demonstrated that the antiepileptic drug carbamazepine can be taken up from soil and reach the pollen and nectar of zucchini plants, highlighting the potential for these contaminants to enter food webs and impact honeybees (*Apis mellifera*) and other foragers.

Insect pollinators are a diverse group that includes moths, butterflies, flies, beetles, and-perhaps-most notably bees. In the UK alone, insect pollination is valued at 0.6 billion.^26^ Although there are many species of bee, *A. mellifera* has been reported as one of the most influential pollinators within agriculture globally (13% of plant species^27^) making use of mass flowering crops and wild plants in agricultural land as a foraging resource.^28^ In the UK alone there has been a 33% decline in pollinators from 1980 to 2013.^29^ The contributory causes to these declines are diverse, and include habitat loss, agricultural use pesticides, invasive species, climate change and other chemical contaminants.^30^ Specifically with regards to chemicals, research to date has shown that honeybees as generalist pollinators, forage on a wide variety of host plants that are known to accumulate pollutants in hive products like honey.^31–34^ For example, a department of Environmental Food and Rural Affairs (DEFRA) report found that 99% of honey samples contained insecticides, fungicides, and herbicides. However, chemical residue analysis is typically focused on a limited set of contaminants, it includes neonicotinoids such as thiamethoxam (<0.04 ng/g ww), clothianidin (0.21–0.77 ng/g), and imidacloprid (0.1–2.85 ng/g),^35^ as well as veterinary antibiotics such as oxytetracycline (22–235 μg/kg ww), doxycline (22–335 μg/kg), and tetracycline (13–900 μg/kg).^36^

The absence of a clear risk assessment framework, comparable to that seen for pesticides, means that actual risks of CECs to native biodiversity are unknown,^37^ despite their increasingly reported presence in agricultural systems. A prerequisite of this is an assessment of exposure and an improved understanding of the presence of CECs in stored hive products. In this study, we assess pharmaceutical residues and other CECs in honey using nontargeted analytical techniques, to semiquantify contaminants in honey from hives located in agricultural landscapes dominated by arable farming. We hypothesized that CECs may contaminate honey through systemic translocation in crops following the use of biosolids and animal manures as fertilizers. This research will establish an important foundation for understanding the exposure of pollinators to these chemicals in real-world agricultural systems, providing a basis for future studies of the toxicological risks associated with such exposures.

## 2 Materials and Methods

### 2.1 Study Details

Honey represents a stored hive product derived from flowering plant nectar sources. For hives located in arable agricultural dominated landscapes this will include crop species (e.g., oilseed rape, field bean), as well as wild plants including those found in close association with cropped fields, e.g., wildflower field margins. We sourced honey samples from 19 hives collected in 2022 by citizen scientist beekeepers as part of the UK National Honey Monitoring Scheme.^38^ Each sample was collected from recently laid down storage comb as opposed to being spun at the end of the season for the purposes of human consumption. Direct sampling from the hive comb avoids dilution of residues across the entire season when harvested for human consumption. Samples were selected from hives located within landscapes with greater than 70% arable cover within <2 km of the hive itself. While *A. mellifera* (honeybees) can forage more than 2 km from hives, the average foraging distance of honeybees is likely closer to 1.5 km from hives.^39^ Moreover, honey is typically considered sterile in nature with minimal microorganisms,^40^ thus removing any concerns in regard to biotic dissipation processes during the time frame between sampling and extraction. Samples were initially stored under ambient conditions in a dark room but after selection were refrigerated until analyses. All honey samples were provided voluntarily by UK beekeepers as part of a citizen-science monitoring program. No experimental procedures involving live animals were conducted, and therefore, institutional animal ethics approval was not required.

### 2.2 Chemicals and Reagents

All chemicals used in this study were of the highest available purity (≥98%). The following compounds were purchased from the indicated suppliers: acetonitrile (VWR, Germany); atrazine (LGC, U.K.); carbamazepine (Sigma-Aldrich); clotrimazole (Sigma-Aldrich) and its internal standard clotrimazole-*d*_5_ (LGC); cyclophosphamide (LGC); diazinon (LGC), diazinon-*d*_10_ (LGC), diclofenac (LGC), diclofenac-*d*_4_ (LGC), enrofloxacin (LGC), enrofloxacin-*d*_5_ (TRC, Canada); lamotrigine (LGC), lincomycin (TRC), and lincomycin-*d*_3_ (LGC); ofloxacin (LGC), ofloxacin-*d*_8_ (Sigma-Aldrich); oxytetracycline (LGC), oxytetracycline-*d*_6_ (LGC); robenidine hydrochloride (LGC), robenidine hydrochloride-*d*_6_ (LGC); sulphamethoxazole (BioVision), sulphamethoxazole-*d*_4_ (LGC); triclosan (LGC), triclosan-*d*_3_ (LGC); trimethoprim (LGC), trimethoprim-*d*_3_ (LGC); and tylosin (LGC). QuECHERS AOAC 2007.01 were purchased from Thermo Scientific and contained anhydrous (MgSO_4_), NaOAc, Primary Secondary Amine (PSA), and C18, and graphitized carbon black.

### 2.3 Extraction

A previously published QuECHERS protocol was modified and followed for the extraction of CECs from *A. mellifera* honey samples, for full details please see Tette et al.^41^ Three replicate samples were analyzed from each of the 19 hives. In brief, 2 g of honey was weighed into a 15 mL Falcon tube and 150 μL of 0.1 μg/mL of deuterated internal standards were added. Eight milliliters of 5% phosphoric acid in acetonitrile and extraction salts (MgSO_4_ and NaOAc) were added, and the sample was vortexed for 2 min. Samples were sonicated at room temperature for 10 min prior to centrifuging at 2500 rpm for 10 min. Clean up sorbents (primary secondary amine, C18, and graphitized black carbon) were added to the solution and pellet prior to decanting and evaporating 8 mL of extract solution to dryness using a GeneVac—25 °C (medium boiling point), (Biopharma, EZ-2). Samples were reconstituted in 10% methanol with deionized water prior to filtering using a 0.2 μm Nylon filter. Samples were refrigerated prior to analyses. Four controls were analyzed with extracted alongside the honey samples, these included a laboratory blank (solvent only) (*n* = 1), 2 g of sucrose (*n* = 1), 2 g of fructose (*n* = 1), and QuECHERS with blank solvent (*n* = 1). The moisture content of honey was ascertained for wet weight–dry weight concentration conversations; please see SI Text Section A and SI Table 1 for details.

### 2.4 Analyses

High-resolution mass spectrometry (HR-MS) was used to identify the chemical fingerprints contained within *A. mellifera* honey samples (Thermo Scientific Vanquish—Exploris). In brief, a reversed phase chromatographic gradient was used which comprised a Waters Acquity HST_3_ column (2.18 μm pore size, 1.5 mm diameter, and 100 mm in length), and separation was achieved using 0.1% formic acid and 1 mM ammonium formate in acetonitrile (mobile phase B), 0.1% formic acid, and 1 mM ammonium formate in ultra purified water (mobile phase A). The gradient was 15 min in duration and contained 0–80, 80–99, 99.1, and 1% mobile phase B at 0.5–8, 8.5–9.5, 9.5–11.5, 11.5–12, and 12–15 min, respectively. The generic mass spectrometry parameters included temperatures of 320 and 350 °C for the ion transfer tube and vaporizer, respectively, and inert gases at 1, 10, and 50 for sweep, auxiliary, and sheath, respectively (nitrogen). Moreover, the ionization methodology that was utilized was ESI; generally it is accepted that pharmaceutical ionization performs best under this methodology. The methodological details included a scan range of 100–1000 Da with 20 scans per second, Multidimensional Ionization and Partitioning (MIPS), dynamic exclusion, and ddMS^2^.

### 2.5 Data Processing and Tentative Identification

Mass spectra were processed using Compound Discoverer (Thermo Scientific, v3.3). Acquired spectra were further analyzed using m/zCloud, the NORMAN SusDat database, and ChemSpider to identify the chemical entities present in the honey samples.^42–44^ Each chemical entity was assigned a confidence level, which was dependent on the database used in the analysis. For example, predictions obtained from mZCloud produced confidences of Level 2a (±0.3 min retention time (RT) ±5 Da, MS/MS comparisons, >70% best match (top hit)) and Level 2b comprised ChemSpider and the Suspect Norman pharmaceutical database (±0.5 min predicted RT, ±5 Da, MS/MS comparisons (2–3 product ions—DDA)).^45^ Confidence ratings were assigned using Schymanski et al.,^45^ in brief Level 1 was achieved with MS, MS/MS comparisons, RT comparisons; Level 2a was achieved when MS, MS/MS to library MS^2^ data (calculated interpretation), Level 2b MS, MS/MS to experimental data (manual interpretation), Level 3 included just MS, MS/MS to experimental data, and Level 4 is Q1 mass (5 ppm) with isotopic/adduct calculations.

To increase confidence of reported chemical entities and to further validate their presence in hives, retention time (RT) predictions and spectral MS/MS comparisons were employed (see SI Text Section B for further details).

### 2.6 Semiquantitative Analyses

Five calibration standards containing deuterated internal standards (IS) (10% methanol) were utilized for semiquantification work. Suspect chemicals were assigned to the most appropriate IS or parent standard, this was done via matching Log IE (ionization efficiency) values, RTs, Log *p*, and molecular weight. An elucidation distance value (<0.5 excellent match < 0.5–1 good match ≥ 1 weak match) was calculated (SI eq 1). This calculation provides insights into the viability of the presented quantified data. Log IE values were calculated as per Liigand et al.^46^ The response factor was calculated by dividing the peak area of a suspect analyte by the concentration of the internal standard or parent. The relative response factor of a chemical sharing similar properties (i.e., internal standard or parent analytical standards) was then used to correct the peak area of the suspect chemical. Semiquantification was achieved using an adjusted equation (SI eq 2), which simplified the approach devised and validated via Aalizadeh et al.,^47^ please see SI Text Section B for details. All analytical standard concentrations were validated against themselves, and the computed fold error was 1.14. Thus, demonstrating a pragmatic approach to semiquantify CECs using nontarget screening. See eq 2 for details (SI eq 2).

### 2.7 Comparisons to Control Matrices

To eliminate false positives, interfering peaks, and potential contamination, strict comparisons were made to a range of control matrices, including blanks, QuEChERS extracts, shop-bought honey, glucose, and fructose. Given the complexity of honey as a matrix, rigorous quality control was essential to ensure accurate reporting of CECs in hive samples. Chemical features detected in shop-bought honey were excluded from the final data set to avoid misattributing background contamination as hive-specific exposure. Compounds that only met Level 3 identification criteria (i.e., multiple probable structural matches) and appeared in 40% or more of the control samples were also removed from further analysis. To semiquantify and accurately assess CEC presence relative to the percentage of arable land (<2 km from the hive), only high-confidence matches were included. Although lower-confidence chemicals were excluded for quality assurance, their potential environmental presence should not be dismissed.

### 2.8 Statistical Analysis

Statistical analyses were conducted using Python in a Jupyter environment, packages included numpy, pandas, matplotlib, seaborn, statsmodels, scipy.stats, sklearn, and lowess for relationship assessments between the proportion of arable land cover within 2 km of each hive and the number of chemicals (active substances) detected. Land cover was quantified using the UKCEH Landcover Map 2022 using 25m rasterized land cover parcels^48^ and were assessed within ArcGIS. Generalized linear modeling was used to examine these associations, with a focus on continuous but over dispersed response data. For this, a Tweedie generalized linear model was utilized using the gllTMB framework,^49^ such statistical evaluations were selected due to the count-like and right skewed nature of the chemical occurrence data.

In addition to the full continuous model, hives were grouped into percentage arable land bands (70–75, 75–80, 80–85, and 85–90%) to allow summary-level visualization and regression at ecologically meaningful thresholds of land-use intensity. To capture potential nonlinear patterns in the relationship between arable land cover and contaminant occurrence, locally estimated scatterplot smoothing (LOESS) was applied to the banded averages.^50^ Mean number of chemicals per grouped arable land bands and the associated standard error of the mean (SEM) were visualized. Model performance was evaluated using *R* ^2^ and adjusted *R* ^2^ values, while significance of linear terms was determined using *p*-values for slope coefficients (based on Pearson’s correlation and linear model outputs). All statistical thresholds were set at α = 0.05.

## 3 Results and Discussion

### 3.1 Nontarget Screening (NTS): Contaminants of Emerging Concern in Honey

CECs were detected in all of the assessed samples (see SI Tables 2 and 3). Following data cleaning to remove chemical entities detected in the controls, RT predictions/filtering, and MS/MS comparisons, a total of 121 chemical features (Levels 3–1) were identified across all samples (SI Tables 2 and 3). On average, 6.8 (±3.01) (standard error (SE)) chemical features were identified per hive (see SI Table 3).

Human-use pharmaceuticals accounted for the largest proportion of detected CECs, representing 64% of the total, followed by industrial chemicals (10%), agrochemicals (9%), surfactants (8%), and plasticizers (5%) (Figure 1) (Level 3–1, please see SI Table 2 for Norman Susdat matches with RT matching, Level 3). While the presence of CECs in the natural environment is relatively well documented, their occurrence in food products such as honey remains poorly understood. In terms of frequency across hives, the top detected CECs were: thionazin (47.4%), *N*,*N*-demethyldodecylamine-*N*-oxide (42.1%), 4-hydroxypropranolol (21.1%), combretastatin (21.1%), aspirin (21.1%), styrene oxide (21.1%), warfarin alcohol metabolite (15.8%), nuarimol (15.8%), and sodium lauroyl sarcosinate (15.8%) (Figure 2). While 4-aminobenzoic acid is of a natural origin, it also possess notable anthropogenic uses (i.e., an intermediate in pharmaceuticals, dyes, and UV filters/sunscreens).^51^ Consequently, their natural occurrence complicates attribution to human activity, and their widespread anthropogenic use and environmental release justify further investigation of these chemicals as potential CECs.

**Figure 1:**
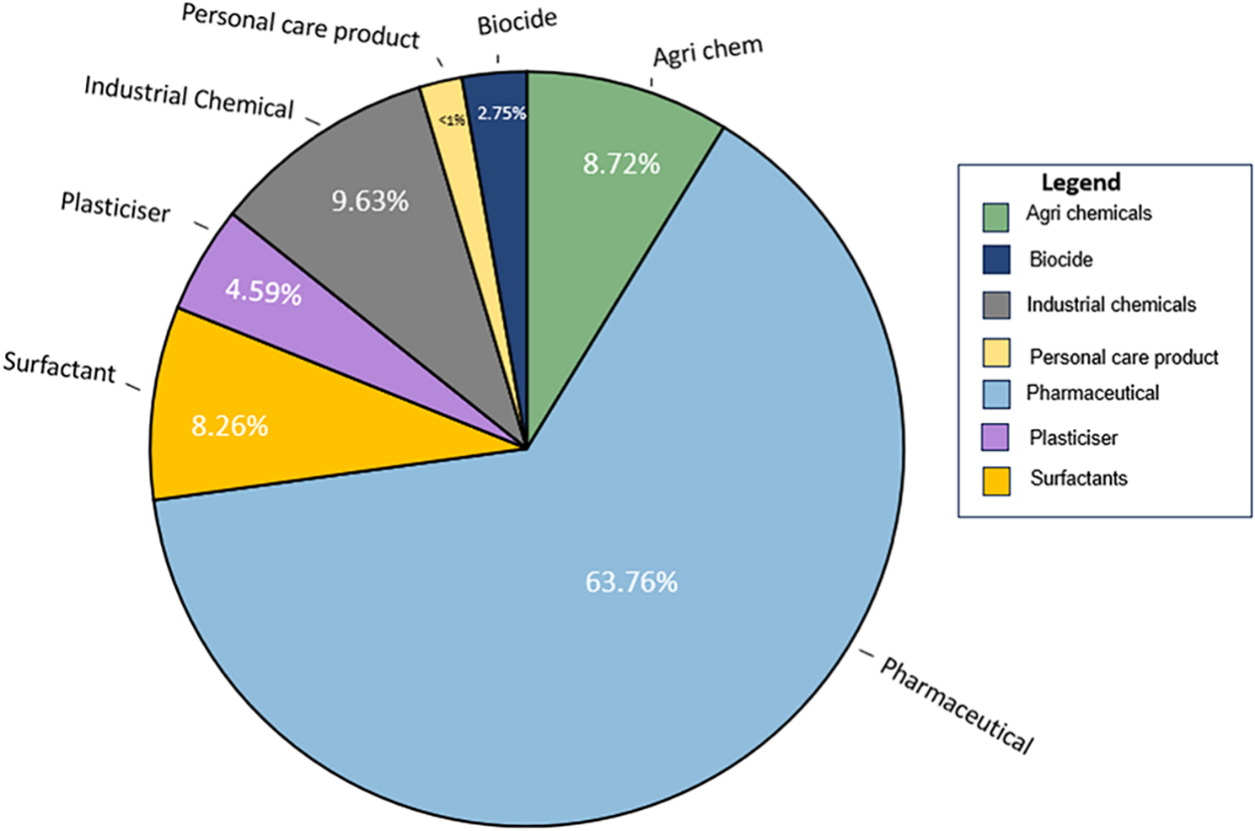
Overview of the detection frequency of the suspect organic chemicals in honey. The presented pie-chart comprises chemicals meeting the Level 3–1 confidence in 19 honey samples with 120 CECs.

**Figure 2:**
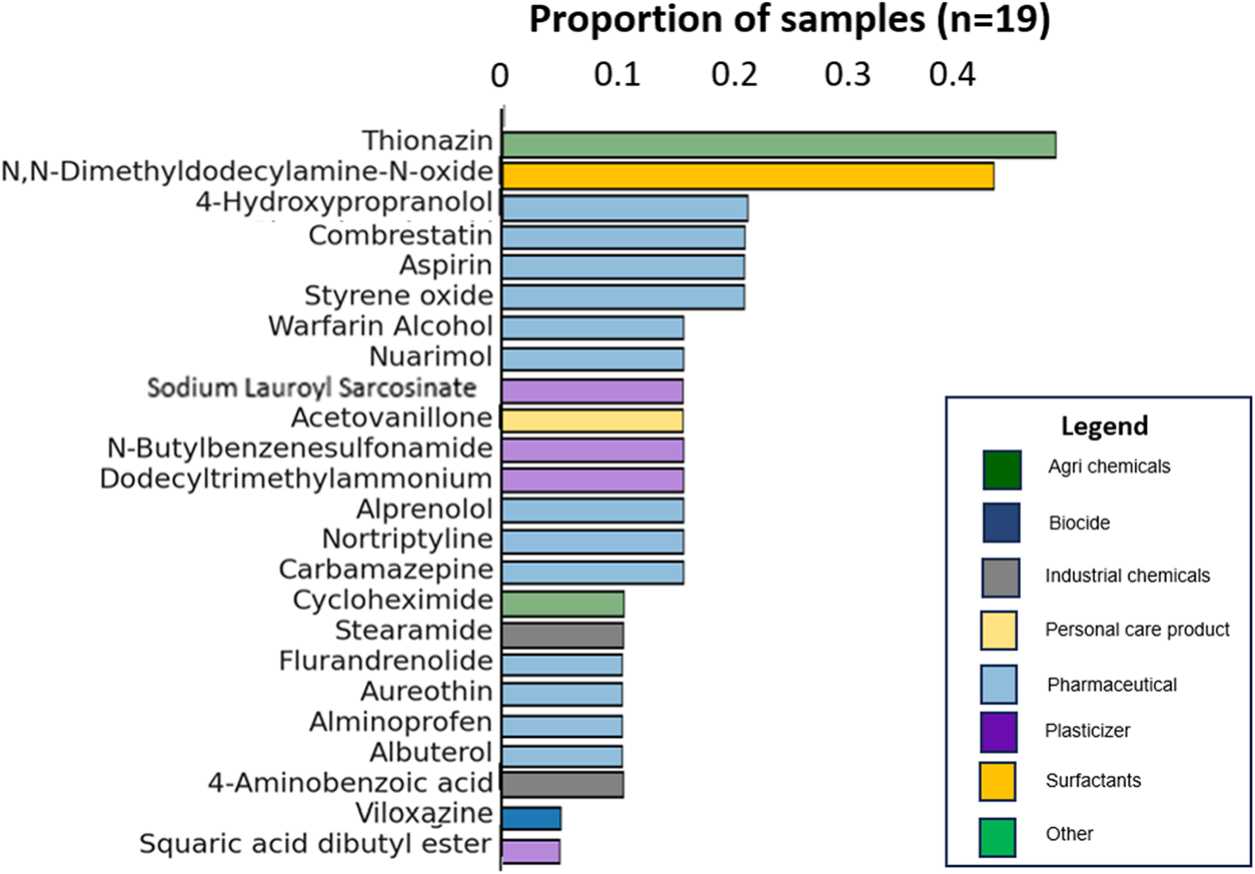
Overview of the most frequently detected (Level 2–1) contaminants of emerging concern in UK honey.

Due to unknown manufacturing/processing techniques, CECs that were present within 66.7% of shop-bought honey were removed from further assessments. However, their presence in the environment or health risks and concerns should not be ruled out. Some of these CECs warrant further investigation and include the agricultural chemicals atrazine (banned herbicide) and nuarimol (fungicide), the pharmaceuticals drespirenone (contraceptive), alverine (antispasmodic), and clofilium (antiarrhhythmic), and the industrial/everyday use chemicals diisooctyl phthalate (plasticizer), triphenylphosphine oxide (flame retardant), erucamide (plastic additive), PEG n5/PPG n4–n8 (polymer), and the UV stabilizer for plastics (4-(dimethylamino)­benzophenone) (SI Table 4). These compounds include agricultural chemicals, flame retardants, rodenticides, surfactants, polymers pharmaceuticals, and their pharmaceutical metabolites, many of which are known to occur in biosolids.^52^ However, their co-occurrence and fate across the soil–plant–hive continuum has received little attention.^53^

The previous lack of detection of a wide range of CECs, including pharmaceuticals, in honey, can be attributed to several factors. Most notably, these compounds were simply not targeted in the analyses. Unlike the well-documented contamination from agriculturally applied pesticides, there was little expectation of a plausible exposure pathway. It was only after the discovery that plants can systemically absorb soil residues from biosolid-fertilized land that a potential route of exposure for insect pollinators via contaminated nectar or pollen was recognized.^8,25^ This exposure pathway is broadly comparable to that of systemic insecticides, such as neonicotinoids, which have been implicated in large-scale declines of pollinator populations.^54,55^

### 3.2 Semiquantified Data—Higher Confidence

Semiquantified concentrations were predicted for all thirty-eight chemicals identified to the highest precision, i.e., a confidence level of 2a and 2b (SI Table 5).^45^ These were selected on the basis of their highest precision. Concentrations of active chemicals in hives were identified to range on average between 0.5 and 582.3 ng/g (dw) (*n* = 18) (CECs—Pharmaceuticals). When ranked by highest detected concentration (dry weight; dw) across all assessed honey samples, the top 25% of chemicals were: aspirin (1649.1 ng/g) > flurandrenolide (1357.03 ng/g) > cycloheximide (575.1 ng/g) > dimethyl sebacate (516.51 ng/g) > azoxystrobin (487.7 ng/g) > methyldopa (198.02 ng/g) > 2,3-diphenylpyrazine (118.3 ng/g) > ibuprofen (11.5 ng/g). These contaminants of emerging concern (CECs) represent diverse chemical classes, including human-use pharmaceuticals (NSAIDs, anti-inflammatories, and antihypertensives), as well as agrochemicals and industrial compounds. As a comparison, the most frequently detected agricultural pesticide residues from honey samples originating from arable agricultural land (80 samples collected in 2021) were the fungicide azoxystrobin (average = 1.37; SE = 0.77; max = 61.6 ng/g w/w), and the insecticides Tau-fluvalinate (average = 2.40; SE = 0.43; max = 25.8 ng/g w/w) and Esfenvalerate (average = 0.22; SE = 0.22; max = 18.0 ng/g w/w).^38^

The detection and quantification of a wide range of CECs, including but not limited to pesticides, are not unexpected. While environmental presence is often linked to usage and exposure, it is also shaped by a compound’s physicochemical properties and environmental fate. Although the estimated global use of pesticides exceeds that of pharmaceuticals (approximately 3 teragrams of pesticides versus 1.9 teragrams of pharmaceuticals (assuming a Defined Daily Dose of 500 mg)) many additional, often undocumented, sources of pharmaceutical emissions may contribute to their disproportionate environmental presence.^56,57^ These include limited metabolic breakdown in humans and animals, and inefficient removal during wastewater treatment.^24,58,59^ Momentarily it remains difficult to compare in-plant concentrations of CECs to the concentrations detected in honey; however, if we consider other contaminant classes who share similar terrestrial fate parameters, a decrease in concentration from fruit is to be expected as a result of further in-plant barriers and prolonged environmental fate processes.^22^

On average (across all hives), the top ten CECs in hives presented a differing hierarchy and included flurandrenolide (582.3 ± 28.4 ng/g) > aspirin (358.2 ± 16.7 ng/g) > dimethyl sebacate (89.8 ± 51.9 ng/g) > methyldopa (123.4 ± 25.2 ng/g) > 2,3-diphenylpyrazine (80 ± 398.3 ng/g) > carbamazepine (70.8 ± 27.2 ng/g) > valomciclovir (63.7 ± 5.5 ng/g) > ibuprofen (28.8 ± 28.9 ng/g) > sulphadiazine (21.9 ± 20.5 ng/g) > trimethoprim (20 ± 3.2 ng/g) on a dry weight basis (Figure 3). The high concentrations and co-occurrence of sulphadiazine and trimethoprim (Figure 3) is likely due to their routine coadministration, particularly in sows and other farmed animals.^60^ The presence of these antibiotics was not unexpected, as they have previously been detected in honey—appearing in 3.2% of 215 samples.^61^ However, the concentrations observed in our study were lower than those reported in earlier research, which ranged from 32.7 to 116.9 ng/g for sulfonamides and 24 to 29.2 ng/g for trimethoprim.^62,63^ Although antibiotics were historically used in beekeeping to treat bacterial infections such as foulbrood, this practice is no longer permitted in the UK.^64^ Furthermore, metadata collected by the National Honey Monitoring Scheme, which includes beekeeper reports on diseases and veterinary treatments, did not indicate the use of antibiotics in any of the sampled hives. This suggests alternative exposure routes, likely from surrounding agricultural practices, such as the application of manure to land. Inadvertent exposure to antibiotics raises significant concerns for bee health as antibiotics can influence the viability of their gut microbiomes which play an important role as a first line of immune defense for bees.^64,65^ Existing evidence of the role of other environmental contaminants on gut microbiomes composition suggests that such effects may have significant implications for the viability of honeybee and potentially wild bee populations.^65–67^

**Figure 3:**
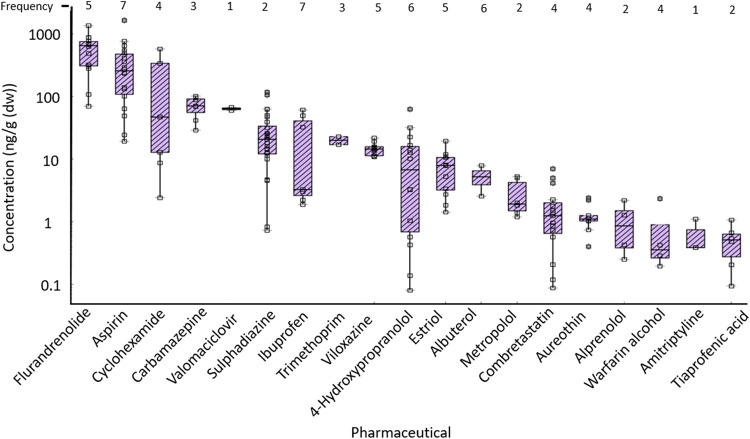
Semiquantitative concentrations of pharmaceuticals in UK honey (Levels 3–1). Values placed on top of the box and whisker plot denote the frequency of detection (meeting the criteria of 66.7% presence in samples). The identification of carbamazepine and trimethoprim were of the Level 1.

### 3.3 Human-Use Pharmaceuticals

The presence of antibiotics in honey in areas with high arable cover is unsurprising given the use of antibiotics to support livestock production and manure/slurry application to land, providing a potential exposure route to foraging bees. However, CECs also detected in honey included medicines of human use such as anticonvulsants, NSAIDs, and antivirals. Semiquantitative analysis predicted a total mass of 5440 ng of pharmaceutically active chemicals across all of the hives combined. As shown in Figure 1, human-use pharmaceuticals (Level 3–1) comprised the most frequently detected class of chemicals in our samples.

Assigned to Level 2 confidence, 52.6% chemical entities were identified as human pharmaceuticals or their metabolites (e.g., gabapentin lactam). However, therapeutic classes reported within this group were diverse, with no clear dominant subgroup. The absence of dominant classes among Level 2b–1 confidence identification is likely due to the limited number of compounds detected at this confidence level. This limitation reflects the analytical challenges associated with compound extraction, detection, and data processing as well as the inherent variability and complexity of CECs in the environment particularly those originating from sources such as biosolids. For example, frequency by pharmaceutical class at Level 3–2 data demonstrated the following hierarchy NSAIDs (*n* = 7) > antibiotics (*n* = 7) > β-blockers (*n* = 7) > antidepressants (*n* = 5) anticonvulsants (*n* = 2).

Among the identified human pharmaceuticals, carbamazepine, ibuprofen, and gemfibrozil are well-known for their environmental persistence; with DT_50_ values in soils reported to range between 170 and 330 days, 6.1 days, and 10.7–14.3 days, respectively.^68,69^ Carbamazepine, gemfibrozil, and ibuprofen have all been previously identified to accumulate to higher plant organs such as fruits (*Solanum lycopersicum*) or grain (*Zea mays*).^20,70^ Moreover, carbamazepine, ibuprofen, gemfibrozil, and trimethoprim have all been previously observed to accumulate in soybean (bean <0.17 ng/g dw carbamazepine), cucumber fruit (0.25–5 ng/g dw ibuprofen), tomato fruits (<0.1 ng/g dw gemfibrozil), and tomato fruits (<0.43 ng/g dw trimethoprim) following soil amendment with biosolids or manure.^71,72^ Evidenced accumulative capacity of these CECs in higher plant organs suggests a potential exposure route for the contamination of honey observed in this study.

Comparatively, very little literature exists regarding the fate or uptake of flurandrenolide, aspirin, valomciclovir (an antiviral similar to that of acyclovir), propranolol, or viloxazine in plants despite the relatively high predicted concentrations in honey samples analyzed as part of this study (SI Tables 3 and 5). Lesser research pharmaceuticals were also identified in honey samples using the NORMAN suspect database (SI Table 4) including viloxazine, valomciclovir (an antiviral similar to that of acyclovir), alprenolol, and amitriptyline. These reported detections highlight our understanding of pharmaceutical presence in the environment, and associated food chain transfer is still in its infancy and supports the notion and requirement for improved monitoring studies.

### 3.4 Potential Exposure Pathways Linked to Arable Cover

Agricultural management practices, particularly the use of organic amendments such as biosolids, clearly influence the environmental distribution of CECs and may significantly contribute to the chemical exposures observed in pollinators. Biosolids, commonly applied to arable land as part of nutrient recycling strategies, have been shown to serve as a key pathway for the introduction of human pharmaceuticals into agri-ecosystems.

Several CECs previously reported in biosolid-amended soils and solid wastes, including carbamazepine, salicylic acid, ibuprofen, and stearic acid,^16^ were also among the most frequently detected compounds in honey. Furthermore, a significant positive correlation was identified between the proportion of arable land cover (grouped into bands—0–75, 75–80, 80–85, and 85–90%) and the number of active chemicals detected per hive (regardless of confidence level classification, Level 2–3; total *n* = 121) (β = 0.0589 ± 0.0258 SE, *z* = 2.28, *p* = 0.022) (Figure 4). This finding highlights the influence of land-use intensity on environmental CEC prevalence and indicates that pollinators located in areas with greater arable land cover are at a higher risk of chemical exposure. Notably, hives within areas with 85–90% arable cover (within a 2 km radius) showed markedly different levels of contamination: some exhibited an average of 4.3 ± 1.9 CECs, while others reached up to 13.5 ± 12.9 CECs. This variability supports the understanding that pollinators are subject to diffuse and complex chemical exposures linked to land-use practices and emphasizes the need for landscape-scale, multichemical risk assessments. Importantly, the majority (64%) of the detected chemicals in this data set were human-use pharmaceuticals, with no known agricultural or veterinary applications. This strengthens the hypothesis that biosolid application, rather than direct agrochemical use, is a primary contributor to pharmaceutical residues detected in honey, particularly in high-arable landscapes.

**Figure 4:**
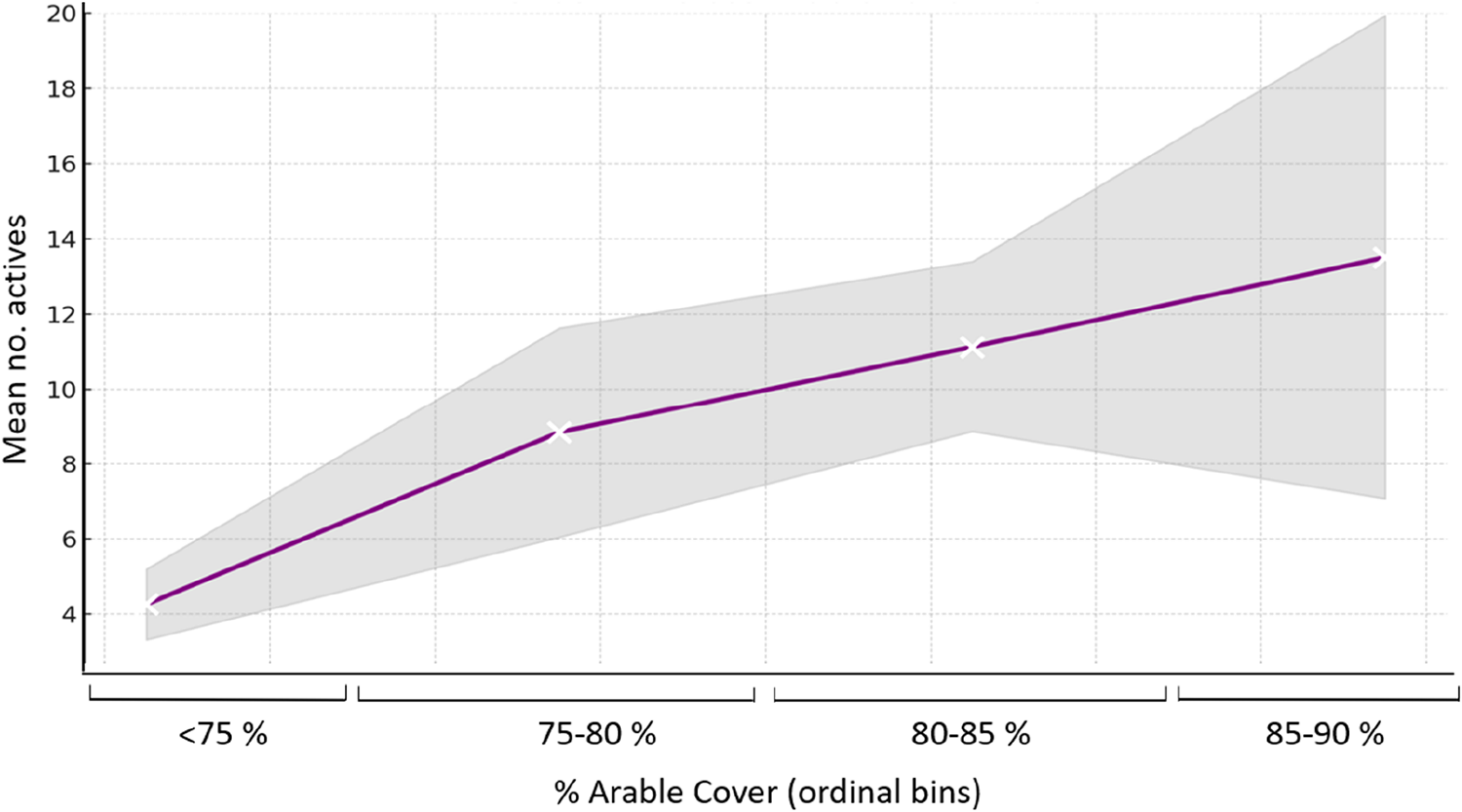
Relationship between the percentage of arable land within 2 km of honeybee hives and the number of active substances detected in honey (Level 3–1). LOES trend for CECs in hives across banned arable land cover (0–75, 75–80, 80–85, and 85–90%). No. of actives refers to co-occurring CECs (e.g., pharmaceuticals, agrichemicals, industrial chemicals etc.). The identification of carbamazepine and trimethoprim were of the Level 1.

### 3.5 Environmental Significance and Risk

The unintentional release of CECs into agricultural environments is well documented.^73–76^ However, our study represents the first broad screening effort to identify and semiquantitatively assess a wide range of CEC classes in beehives across regions with contrasting but intensive percentages of arable cover.^76–78^ Our study revealed the widespread presence of CECs in the limited number of UK honey samples analyzed, but these findings warrant further investigation as they could have important implications for global change. While pesticide impacts on bees are relatively well studied, far less is known about the effects of other environmental contaminants on forager health, particularly at environmentally relevant concentrations and exposure durations.^25^ This concern is heightened by our finding that human pharmaceuticals make up the largest fraction of identified CECs in honey, with concentrations ranging from 0.5 ± 0.3 to 582.3 ng/g (dw), alongside frequently detected veterinary antibiotics.

Previous research has demonstrated the potential adverse effects of these compounds on bee health. For example, oxytetracycline, a veterinary antibiotic formerly used directly in hives for therapeutic purposes, has been shown to increase the susceptibility of bees to the ecotoxicological effects of pesticides.^79^ Similarly, 450 μg/mL of tetracycline treatment has been identified to alter bee gut microbiomes, shifting structures, and affecting/contributing toward, multidrug resistance transport, metabolism, immunocompetency, and pest defense.^80,81^ Momentarily, little is known regarding the influence of antibiotics on bee health/functioning; previous exposure concentrations were elevated in comparison to the concentrations reported here in honey (273.5 ± 28.8 ng/g dw) (summed—sulphadiazine, and trimethoprim). However, such effects are likely to increase the susceptibility of bees to other contaminants, such as fungicides and pesticides and contribute toward the decline in nutrition levels, behavioral activity (foraging), and hive augmentation. Antibiotics present in pollen, nectar, and hives can promote the development of antimicrobial resistance (AMR).^82^ For instance, Piva et al.^83^ detected resistance genes in 12 of 48 hive samples, many unrelated to beekeeping practices and more likely linked to human or veterinary antibiotic use. The widespread detection of antibiotics (*n* = 5, 26.32% of hives) detected in this study suggests important implications for bee health and the development of AMR more broadly.

Moving forward to accurately assess the risks CECs pose to bees; it is essential to determine accurate exposure concentrations in key matrices (e.g., nectar) but also to be able to relate this to known toxicities; information which is currently absent for most of these compounds. In addition to the urgent need to consider the toxicity of contaminants to bees beyond agricultural use chemicals (e.g., pesticides), it is important to highlight that current regulatory approaches typically focus on standard end points like NOED, LD_50_’s derived from dose–response range-finding methods. These approaches often overlook the effects of chronic exposure to environmentally relevant concentrations, which may lead to subtle yet significant behavioral and physiological changes, such as learning, foraging, feeding, memory, and generic functioning.^84–86^ Even for well-regulated pesticides, the risk posed by long-term low-level exposure have often been hard to predict.^1^ As previous research has demonstrated the potential for trace levels of CECs in the environment, including human pharmaceuticals, to impact on behavioral and physiological end points in wildlife, it is imperative that this is considered when evaluating the risk CECs pose to honeybees.^87^

Moreover, the potential risk to consumers from the ingestion of contaminated honey remains largely unexplored; in addition, it is possible that the associated risks differ in both local and regional scale production (watering, additives; chemical use, agricultural practices). Unlike pesticides, for which dietary exposure guidelines exist, there are currently no established regulatory thresholds for most CECs in food products, including honey. This creates a significant knowledge gap in food safety assessments, particularly given the complex nature of chemical mixtures. Previous risk assessments examining CECs in contaminated produce, such as vegetables irrigated with reclaimed wastewater or grown in biosolid-amended soils, have demonstrated that hazard quotients (HQs) for mixtures of CECs may exceed 1,^25^ indicating potential health risks. Pharmaceuticals and personal care products detected in honey retain their biological activity and were not intended for oral exposure through food; understanding their potential chronic effects, especially among vulnerable populations such as children and those with compromised health, is critical. As honey is commonly consumed in raw or minimally processed forms, further research is urgently needed to assess the dietary exposure, bioavailability, and long-term health implications of CECs in honey.

## Supplementary Material



## Notes

Methodological details into the identification of CECs using external spectral libraries as well as the mechanisms used for semiquantification using parent and internal standard analytes; raw data is also contained within, which includes CECs identified in hives at varying land-use percentages; nontargeted data is separated into varying confidence levels, all data was included speculative to higher accuracy to facilitate further exploration and research; moreover, semiquantified and quantified data is included; finally, chemicals identified in shop-bought honey were also included, while this was a strict control measure due to a analytically challenging matrix, it also demonstrates CECs in honey available for purchase to the general public (PDF)

## Abbreviations

CECs: compounds of emerging concern
log IE: ionization efficiency
NOED: no observed effect dose
LOESS: locally estimated scatterplot smoothing
SEM: standard error mean
HR-MS: high-resolution mass spectrometry
dw: dry weight
LD_50_: lethal dose to 50% of the population
DT_50_: half life
NSAIDs: nonsteroidal anti-inflammatory drugs
HQ: hazard quotient
AMR: antimicrobial resistance
