# Supplementary material for: Presence of Emerging Contaminants in UK Honey—Human Pharmaceuticals a Concern for Honeybees?
Source: J Agric Food Chem. 2026 Mar 6;74(26):20251–60. doi: 10.1021/acs.jafc.5c10414 (PMC13352592; doi:10.1021/acs.jafc.5c10414)
Supplement: Supplementary file 1 [file jf5c10414_si_001.pdf]

Supporting Information

The Presence of Emerging Contaminants in UK Honey - Human Pharmaceuticals a Concern for Honeybees?

Authors: John Nightingale[1*], Ben A. Woodcock[2], Narmin Garazade[1], Richard F. Pywell[2] and Laura J. Carter[1,3]

Affiliations departments and addresses

[1] School of Geography, The University of Leeds, Leeds, LS9 2JT, UK

[2] UK Centre for Ecology & Hydrology, Crowmarsh Gifford, Wallingford, OX10 8BB, UK

[3] Water@leeds, The University of Leeds, Leeds, LS9 2JT, UK

Corresponding author: John Nightingale

ORCID ID's: Ben Woodcock (0000-0003-0300-9951), John Nightingale (0000-0002-8690-0303), Laura Carter (0000-0002-1146-7920), Richard Pywell (0000-0001-6431-9959), Narmin Garazade (0000-0002-8377-1913)

Co-author emails: l.j.Carter@Leeds.ac.uk, rfp@ceh.ac.uk, bawood@ceh.ac.uk, sphc7386@leeds.ac.uk

Table of contents

Section	Description	Page
SI Text Section A	Moisture content determination	S-3
SI Text Section B	Confidence level assignment, RT prediction, and elucidation metrics	S-3
SI Equations (SI Eq. 1–2)	Elucidation distance and semi quantification	S-4
SI Table 1	Moisture contents of honey samples	S-5
SI Table 2	CECs identified in GB beehives to level 3 using the Norman Susdat dataset and RT predictions/evaluations.	S-6-19
SI Table 3	CECs identified in a range of beehives at a higher confidence interval levels 3-1.	S-20-26
SI Table 4	Contaminants present in shop bought honey (control) - Level 3-2a.	S-27-33
SI Table 5	Semi Quantified concentrations of pharmaceuticals present in UK beehives (honey) (Level 2-1).	S-34

SI Text Section A

Moisture content was determined using a handheld refractometer (HMX-1, Honey Moisture, ATC; Sigma-Aldrich, UK) following the manufacturer's instructions [1]. In brief, a small drop of homogenised honey was placed on the prism surface, ensuring the sample was free of air bubbles and equilibrated to room temperature. The cover plate was closed to form a uniform film, and the moisture percentage was read directly from the instrument's internal scale, which automatically compensates for temperature (ATC).

SI Text Section B

Combined these multiple confidence checks supported reporting to a confidence level of 3 [1]. In a step to provide further confirmation of CECs presence matched RT predictions and spectra from both ChemSpider, and the NORMAN pharmaceutical database [2-3] were compared to varying sources of MS/MS data such as GNSP, NIST, and the NCBI (Level 2b). It was considered that level 3 criteria was met when two product ions matched that of external databases.

RT predictions were achieved using PredRet [4], a QSAR approach utilising previously derived RTs to predict differences in other LC systems. The authors of the QSAR model reported a relative error of 2.6 % in their predictions, predicted RTs had a slightly wider tolerance of ±0.5 minutes to compensate for potential error (0.3 < 0.5 minutes), whilst such tools are deemed accurate, they do introduce a level of inaccuracy, thus to capture all chemicals of interest RT tolerance was broadened slightly which still meets a confidence level of 3. The presented equation (SI Equation 2) utilises the fundamentals set out via Aalizadeh *et al.,* [7], and earlier work via, Oss *et al.,* [8], and Liigan *et al.,* [9-10]. The parameters that remain include LogIE – predicted (present) vs experimental [7], peak area, and known concentration. However, the presented equation removed molecular weight to remove duplicate weighting – it is included already within logIE prediction. Moreover, slope was removed for analytical ease – the NTS method was not developed for targeted work and thus some calibrants performed poorly at the lower concentration ranges. Therefore, slope was replaced with known concentration multiplied by the peak area ratio (intercept ≈ 0).

SI Eq 1:

$$\boldsymbol{Elucidation\ distance} = \sqrt{(x_1 - y_1)^2} + (x_2 - y_2)^2 + (x_3 - y_3)^2 + (x_4 - y_4)^2$$

SI Eq 2:

$$\boldsymbol{Concentration_{analyte}} = Concentration_{Standard\ or\ internal\ standard} \times \frac{Peak\ area\ of\ the\ analyte}{Peak\ area_{Internal\ standard}}) \times 10^{LogIE\ standard} - LogIE_{Suspect}$$

Where: x_1 y_1 **=** *standardised LogIE,* $x_2 - y_2$ *standardised retention time (RT),* $x_3 - y_3$ *standardised molecular weight,* $x_4 - y_4$*, standardised log p and standardisation refers to the correction of a value via the mean and standard deviation.*

SI Table 1: Moisture contents of honey samples.

Percentage of arable cover < 2km of hive	Honey moisture content (%)
71.92	15.6
73.01	19
73.9	16.5
73.94	16
73.96	21
74.37	18
75.13	18
75.15	17.3
75.49	16.5
75.72	16
78.33	16
80.39	17
80.4	16.6
81.2	18.2
83.93	17
84.94	21.8
86.13	16.5
89.63	17
90.98	18

SI Table 2: CECs identified in GB beehives to level 3 using the Norman Susdat dataset and RT predictions/evaluations.

Name	Class	Predicted RT -	Experimental RT	Formula	Mass Deviation [ppm]	Calc. MW	% arable <2 km of hive	No. actives
(S)-MCPG	Pharmaceutical - mGluR Antagonist	4.0075	4.279	C10 H11 N O4	-0.09	209.0688		
[1,1'-Biphenyl]-4,4'-dicarboxylic acid, dimethyl ester	Pharmaceutical - Hepatoprotective Agent	6.8645	6.776	C16 H14 O4	0.53	270.0894		
2,3-Dihydroxybenzoic acid	Pharmaceutical - Iron chelator (natural)	4.461	4.515	C7 H6 O4	-0.2	154.0266		
2',4,4'-Trihydroxychalcone	Pharmaceutical - Flavonoid	7.876	7.915	C15 H12 O4	0.1	256.0736		
Acetovanillone	Flavouring agent	5.485	5.485	C9 H10 O3	-0.3	166.0629		
Adenine	Biological compound - Nucleobase	1.579	1.579	C5 H5 N5	0.15	135.0545		
Aspirin	Pharmaceutical - NSAID	5.168	5.008	C9 H8 O4	-0.26	180.0422		
Benzoic acid	Pharmaceutical - Preservative / Industrial Chemical	5.1405	5.401	C7 H6 O2	-0.18	122.0368	90.98	15
Cinacalcet	Pharmaceutical - Calcimimetic	11.753	11.753	C22 H22 F3 N	-4.98	357.1687		
Combretastatin	Pharmaceutical – Vascular Disrupting Agent	6.18	6.18	C18 H22 O6	0.42	334.1418		
L-Tyrosine	Pharmaceutical - Amino Acid (Nutraceutical)	4.569	4.569	C9 H11 N O3	0.06	181.0739		
Meconin	Natural Product - Methylenedioxyphenyl lactone	5.3745	5.601	C10 H10 O4	-0.24	194.0579		
Styrene oxide	Industrial Chemical - Epoxide Intermediate	5.417	5.395	C8 H8 O	-0.88	120.0574		
Tucaresol	Pharmaceutical - Immunopotentiator	6.921	6.921	C15 H12 O5	-0.4	272.0684		
Xanthine	Pharmaceutical - Purine	2.3665	2.098	C5 H4 N4 O2	-0.12	152.0334		

	Derivative (Metabolite)							
[1,1'-Biphenyl]-4,4'-dicarboxylic acid, dimethyl ester	Pharmaceutical - Hepatoprotective Agent	6.8645	6.776	C16 H14 O4	0.53	270.0894		
2,3-Dihydroxybenzoic acid	Pharmaceutical - Iron chelator (natural)	4.461	4.515	C7 H6 O4	-0.13	154.0266		
2,3-Dimethoxy-5,6-dimethyl-p-benzoquinone	Industrial chemical - Quinone derivative	5.09	5.527	C10 H12 O4	-0.36	196.0735		
2',4,4'-Trihydroxychalcone	Pharmaceutical - Flavonoid	7.876	7.915	C15 H12 O4	0.1	256.0736	89.63	7
Ritobegron	Pharmaceutical - β3-Adrenoceptor Agonist	6.3245	6.536	C21 H27 N O5	-0.7	373.1887		
Styrene oxide	Industrial Chemical - Epoxide Intermediate	5.417	5.395	C8 H8 O	-0.89	120.0574		
Tucaresol	Pharmaceutical - Immunopotentiator	6.921	6.921	C15 H12 O5	-0.4	272.0684		
(S)-3,5-Dihydroxyphenylglycine	Pharmaceutical - mGluR Agonist	3.69	3.411	C8 H9 N O4	-0.19	183.0531		
[1,1'-Biphenyl]-4,4'-dicarboxylic acid, dimethyl ester	Pharmaceutical - Hepatoprotective Agent	6.8645	6.776	C16 H14 O4	0.35	270.0893		
2,3-Dihydroxybenzoic acid	Pharmaceutical - Iron chelator (natural)	4.461	4.515	C7 H6 O4	-0.13	154.0266		
2',4,4'-Trihydroxychalcone	Pharmaceutical - Flavonoid	7.876	7.915	C15 H12 O4	0.1	256.0736		
3-(3,4-Dimethoxyphenyl)propionic acid	Pharmaceutical - Natural product analog	5.244	5.381	C11 H14 O4	-0.32	210.0891		
3,6,9,12,15-Pentaoxatricosan-1-ol	Industrial chemical - Polyether alcohol	6.245	6.291	C18 H38 O6	-0.92	350.2665		
4-(2-Aminophenyl)-4-oxobutanoic acid	Pharmaceutical - Drug intermediate	4.7795	5.241	C10 H11 N O3	0.08	193.0739		
4-Nitrophenylgalactoside	Industrial chemical - Chromogenic substrate	3.9375	4.26	C12 H15 N O8	0.04	301.0798		
Acetovanillone	Flavourant	5.485	5.485	C9 H10 O3	-0.87	166.0629		
Apigenin	Pharmaceutical - Flavonoid	7.941	7.941	C15 H10 O5	0.41	270.0529		
Aspirin	Pharmaceutical - NSAID	5.168	5.008	C9 H8 O4	-0.26	180.0422	86.13	32

Benzoic acid	Pharmaceutical - Preservative / Industrial Chemical	5.1405	5.401	C7 H6 O2	0.02	122.0368
Carbocysteine	Pharmaceutical - Mucolytic	2.848	2.848	C5 H9 N O4 S	0.07	179.0252
Castanospermine	Natural Product - Alkaloid	3.218	3.218	C8 H15 N O4	-0.17	189.1001
Daidzein	Pharmaceutical - Isoflavone (Phytoestrogen)	7.999	7.808	C15 H10 O4	0.62	254.0581
Esculin	Pharmaceutical - Natural Coumarin	4.6095	4.113	C15 H16 O9	-0.66	340.0792
Gemcabene	Pharmaceutical - Lipid-lowering Agent	6.75	6.868	C16 H30 O5	0.52	302.2095
Hydrocinnamic acid	Industrial Chemical - Flavouring Agent	5.413	5.413	C9 H10 O2	-0.09	150.0681
Maltol	Industrial Chemical - Flavour Enhancer	3.748	3.569	C6 H6 O3	-0.14	126.0317
Meconin	Natural Product - Methylenedioxyphenyl lactone	5.3745	5.601	C10 H10 O4	-0.24	194.0579
Methyl salicylate	Pharmaceutical - Lipid-lowering Agent	5.252	4.838	C8 H8 O3	0.03	152.0474
Nanafrocin	Pharmaceutical - Antifungal (Polyene-like)	6.9905	7.082	C16 H14 O6	0.57	302.0792
Oseltamivir	Pharmaceutical - Antiviral (Neuraminidase Inhibitor)	6.996	6.996	C16 H28 N2 O4	0.64	312.2051
Sakuranetin	Natural Product - Flavonoid	6.501	6.501	C16 H14 O5	-0.12	286.0841
Styrene oxide	Industrial Chemical - Epoxide Intermediate	5.417	5.395	C8 H8 O	-0.88	120.0574
Swainsonine	Natural Product - Indolizidine Alkaloid	4.984	5.053	C8 H15 N O3	0.02	173.1052
Tert-Butylhydroquinone	Industrial Chemical - Food Preservative	5.418	5.712	C10 H14 O2	-0.66	166.0993
Tucaresol	Pharmaceutical - Immunopotentiator	6.921	6.921	C15 H12 O5	-0.4	272.0684

Uridine	Pharmaceutical - Nucleoside (Nutritional/Neurological)	2.532	3.07	C9 H12 N2 O6	0.48	244.0697		
Xanthine	Pharmaceutical - Purine Derivative (Metabolite)	2.3665	2.098	C5 H4 N4 O2	-0.12	152.0334		
Zebularine	Pharmaceutical - Nucleoside Analog (Epigenetic modulator)	3.613	3.613	C9 H12 N2 O5	0.56	228.0748		
12-Hydroxydodecanoic acid	Industrial chemical - Fatty acid derivative	8.623	7.85596	C12H24O3		215.1652		
2,3-Dihydroxybenzoic acid	Pharmaceutical - Iron chelator (natural)	4.461	4.68865	C7H6O4		153.0193		
3-(3,4-Dimethoxyphenyl)propionic acid	Pharmaceutical - Natural product analogue	5.244	5.0939	C11H14O4		209.0818		
Acetovanillone	Personal care product /everyday use- Fragrant or Flavouring agent	5.485	5.08154	C9H10O3		165.0555		
Apigenin	Pharmaceutical - Flavonoid	7.941	8.17479	C15H10O5		269.0457		
Aspirin	Pharmaceutical - NSAID	5.168	5.61303	C9H8O4		179.0349		
Benzoic acid	Pharmaceutical - Preservative / Industrial Chemical	5.1405	5.11364	C7H6O2		121.0295		
Daidzein	Pharmaceutical - Isoflavone (Phytoestrogen)	7.999	8.401	C15H10O4		255.0652		
Hydro cinnamic acid	Industrial Chemical - Flavouring Agent	5.413	5.88281	C9H10O2		149.0607		
Phenylacetic acid	Naturally occurring (in honey)	4.987	4.7483	C8H8O2		135.0451		
Styrene oxide	Industrial Chemical - Epoxide Intermediate	5.417	5.58594	C8H8O		165.0557		
Swainsonine	Natural Product - Indolizidine Alkaloid	4.984	4.96104	C8H15NO3		172.0979	86.13	12
(S)-3,5-Dihydroxyphenylglycine	Pharmaceutical - mGluR Agonist	3.69	3.411	C8 H9 N O4	0.06	183.0532	84.94	21
[1,1'-Biphenyl]-4,4'-dicarboxylic acid, dimethyl	Pharmaceutical - Hepatoprotective Agent	6.8645	6.776	C16 H14 O4	0.35	270.0893		

ester						
2,3-Dihydroxybenzoic acid	Pharmaceutical - Iron chelator (natural)	4.461	4.515	C7 H6 O4	-0.14	154.0266
2,3-Dimethoxy-5,6-dimethyl-p-benzoquinone	Industrial chemical - Quinone derivative	5.09	5.527	C10 H12 O4	-0.22	196.0735
2',4,4'-Trihydroxychalcone	Pharmaceutical - Flavonoid	7.876	7.915	C15 H12 O4	0.1	256.0736
3-(3,4-Dimethoxyphenyl)propionic acid	Pharmaceutical - Natural product analogue	5.244	5.381	C11 H14 O4	-0.32	210.0891
3,6,9,12,15-Pentaoxatricosan-1-ol	Industrial chemical - Polyether alcohol	6.245	6.291	C18 H38 O6	-0.92	350.2665
Acetovanillone	Personal care product /everyday use- Fragrant or Flavouring agent	5.485	5.485	C9 H10 O3	-1.14	166.0628
Apigenin	Pharmaceutical - Flavonoid	7.941	7.941	C15 H10 O5	0.41	270.0529
Aspirin	Pharmaceutical - NSAID	5.168	5.008	C9 H8 O4	-0.17	180.0422
Benzoic acid	Pharmaceutical - Preservative / Industrial Chemical	5.1405	5.401	C7 H6 O2	0.02	122.0368
Methyl salicylate	Pharmaceutical - Lipid-lowering Agent	5.252	4.838	C8 H8 O3	-0.22	152.0473
Nanafrocin	Pharmaceutical - Antifungal (Polyene-like)	6.9905	7.082	C16 H14 O6	0.57	302.0792
Oseltamivir	Pharmaceutical - Antiviral (Neuraminidase Inhibitor)	6.996	6.996	C16 H28 N2 O4	0.64	312.2051
Phenylacetic acid	Naturally occurring (in honey)	4.987	5.557	C8 H8 O2	-0.08	136.0524
Sakuranetin	Natural Product - Flavonoid	6.501	6.501	C16 H14 O5	-0.12	286.0841
Styrene oxide	Industrial Chemical - Epoxide Intermediate	5.417	5.395	C8 H8 O	-0.88	120.0574
Swainsonine	Natural Product - Indolizidine Alkaloid	4.984	5.053	C8 H15 N O3	0.11	173.1052
Uridine	Pharmaceutical -	2.532	3.07	C9 H12 N2	0.48	244.0697

	Nucleoside (Nutritional/Neurological)			O6				
Xanthine	Pharmaceutical - Purine Derivative (Metabolite)	2.3665	2.098	C5 H4 N4 O2	-0.12	152.0334		
2,3-Dihydroxybenzoic acid	Pharmaceutical - Iron chelator (natural)	4.461	4.68865	C7H6O4	84.94	153.0193		
3-(3,4-Dimethoxyphenyl)propionic acid	Pharmaceutical - Natural product analogue	5.244	5.0939	C11H14O4	84.94	209.0818		
Acetovanillone	Personal care product /everyday use- Fragrant or Flavouring agent	5.485	5.08154	C9H10O3	84.94	165.0555	84.94	6
Apigenin	Pharmaceutical - Flavonoid	7.941	8.17479	C15H10O5	84.94	269.0457		
Aspirin	Pharmaceutical - NSAID	5.168	5.61303	C9H8O4	84.94	179.0349		
Benzoic acid	Pharmaceutical - Preservative / Industrial Chemical	5.1405	5.11364	C7H6O2	84.94	121.0295		
(S)-3,5-Dihydroxyphenylglycine	Pharmaceutical - mGluR Agonist	3.69	3.411	C8 H9 N O4	-0.15	183.0531		
(S)-MCPG	Pharmaceutical - mGluR Antagonist	4.0075	4.279	C10 H11 N O4	0.07	209.0688		
[1,1'-Biphenyl]-4,4'-dicarboxylic acid, dimethyl ester	Pharmaceutical - Hepatoprotective Agent	6.8645	6.776	C16 H14 O4	0.53	270.0894		
2,3-Dihydroxybenzoic acid	Pharmaceutical - Iron chelator (natural)	4.461	4.515	C7 H6 O4	-0.13	154.0266		
2,3-Dimethoxy-5,6-dimethyl-p-benzoquinone	Industrial chemical - Quinone derivative	5.09	5.527	C10 H12 O4	-0.36	196.0735		
3-(3,4-Dimethoxyphenyl)propionic acid	Pharmaceutical - Natural product analogue	5.244	5.381	C11 H14 O4	-0.8	210.089		
4-Nitrophenylgalactoside	Industrial chemical - Chromogenic substrate	3.9375	4.26	C12 H15 N O8	0.04	301.0798		
Acetovanillone	Personal care product /everyday use- Fragrant or Flavouring agent	5.485	5.485	C9 H10 O3	-0.25	166.063		
Apigenin	Pharmaceutical - Flavonoid	7.941	7.941	C15 H10 O5	0.41	270.0529	83.93	17

Benzoic acid	Pharmaceutical - Preservative / Industrial Chemical	5.1405	5.401	C7 H6 O2	0	122.0368		
Cinacalcet	Pharmaceutical - Calcium sensing receptor agonist	11.753	11.753	C22 H22 F3 N	-4.98	357.1687		
Gemcabene	Pharmaceutical - Lipid-lowering Agent	6.75	6.868	C16 H30 O5	0.52	302.2095		
Isovaleric acid	Industrial Chemical - Fragrance Ingredient	4.6095	4.558	C5 H10 O2	0.29	102.0681		
Methyl salicylate	Pharmaceutical - Lipid-lowering Agent	5.252	4.838	C8 H8 O3	0	152.0473		
Sakuranetin	Natural Product - Flavonoid	6.501	6.501	C16 H14 O5	-0.12	286.0841		
Tucaresol	Pharmaceutical - Immunopotentiator	6.921	6.921	C15 H12 O5	-0.4	272.0684		
2,3-Dihydroxybenzoic acid	Pharmaceutical - Iron chelator (natural)	4.461	4.68865	C7H6O4	81.12	153.0193		
Acetovanillone	Flavouring agent	5.485	5.08154	C9H10O3	81.12	165.0555		
Aspirin	Pharmaceutical - NSAID	5.168	5.61303	C9H8O4	81.12	179.0349		
Benzoic acid	Pharmaceutical - Preservative / Industrial Chemical	5.1405	5.11364	C7H6O2	81.12	121.0295		
L-Tyrosine	Pharmaceutical - Amino Acid (Nutraceutical)	4.569	4.96104	C9H11NO3	81.12	240.0878		
Styrene oxide	Industrial Chemical - Epoxide Intermediate	5.417	5.58594	C8H8O	81.12	165.0557	81.93	
2,3-Dihydroxybenzoic acid	Pharmaceutical - Iron chelator (natural)	4.461	4.68865	C7H6O4	83.93	153.0193		
3-(3,4-Dimethoxyphenyl)propionic acid	Pharmaceutical - Natural product analogue	5.244	5.0939	C11H14O4	83.93	209.0818		
Apigenin	Pharmaceutical - Flavonoid	7.941	8.17479	C15H10O5	83.93	269.0457		
Benzoic acid	Pharmaceutical - Preservative / Industrial Chemical	5.1405	5.11364	C7H6O2	83.93	121.0295	81.12	6
Isovaleric acid	Industrial Chemical -	4.6095	4.68865	C5H10O2	83.93	249.1344		

Phenylacetic acid	Fragrance Ingredient Naturally occurring (in honey)	4.987	4.7483	C8H8O2	83.93	135.0451		
(S)-3,5-Dihydroxyphenylglycine	Pharmaceutical - mGluR Agonist	3.69	3.411	C8 H9 N O4	-0.19	183.0531		
(S)-MCPG	Pharmaceutical - mGluR Antagonist	4.0075	4.279	C10 H11 N O4	-0.09	209.0688		
2,3-Dihydroxybenzoic acid	Pharmaceutical - Iron chelator (natural)	4.461	4.515	C7 H6 O4	-0.13	154.0266		
2,3-Dimethoxy-5,6-dimethyl-p-benzoquinone	Industrial chemical - Quinone derivative	5.09	5.527	C10 H12 O4	-0.02	196.0736		
2',4,4'-Trihydroxychalcone	Pharmaceutical - Flavonoid	7.876	7.915	C15 H12 O4	0.1	256.0736		
3-(3,4-Dimethoxyphenyl)propionic acid	Pharmaceutical - Natural product analog	5.244	5.381	C11 H14 O4	-0.32	210.0891		
4-Nitrophenylgalactoside	Industrial chemical - Chromogenic substrate	3.9375	4.26	C12 H15 N O8	0.04	301.0798		
Aspirin	Pharmaceutical - NSAID	5.168	5.008	C9 H8 O4	-0.26	180.0422		
Benzoic acid	Pharmaceutical - Preservative / Industrial Chemical	5.1405	5.401	C7 H6 O2	0.02	122.0368		
Cinacalcet	Pharmaceutical - Calcimimetic	11.753	11.753	C22 H22 F3 N	-4.98	357.1687		
Gemcabene	Pharmaceutical - Lipid-lowering Agent	6.75	6.868	C16 H30 O5	0.6	302.2095		
Hydrocinnamic acid	Industrial Chemical - Flavouring Agent	5.413	5.413	C9 H10 O2	-0.09	150.0681		
Meconin	Natural Product - Methylenedioxyphenyl lactone	5.3745	5.601	C10 H10 O4	-0.24	194.0579		
Methyl salicylate	Pharmaceutical - Lipid-lowering Agent	5.252	4.838	C8 H8 O3	-0.22	152.0473		
Nanafrocin	Pharmaceutical - Antifungal (Polyene-like)	6.9905	7.082	C16 H14 O6	0.57	302.0792		
Styrene oxide	Industrial Chemical - Epoxide Intermediate	5.417	5.395	C8 H8 O	-0.55	120.0575		
Tucaresol	Pharmaceutical - Immunopotentiator	6.921	6.921	C15 H12 O5	-0.4	272.0684	80.4	18

Zebularine	Pharmaceutical - Nucleoside Analog (Epigenetic modulator)	3.613	3.613	C9 H12 N2 O5	0.56	228.0748		
[1,1'-Biphenyl]-4,4'-dicarboxylic acid, dimethyl ester	Pharmaceutical - Hepatoprotective Agent	6.8645	6.776	C16 H14 O4	0.53	270.0894		
2,3-Dihydroxybenzoic acid	Pharmaceutical - Iron chelator (natural)	4.461	4.515	C7 H6 O4	-0.13	154.0266		
2,3-Dimethoxy-5,6-dimethyl-p-benzoquinone	Industrial chemical - Quinone derivative	5.09	5.527	C10 H12 O4	-0.36	196.0735		
2',4,4'-Trihydroxychalcone	Pharmaceutical - Flavonoid	7.876	7.915	C15 H12 O4	0.1	256.0736		
Ritobegron	Pharmaceutical - β3-Adrenoceptor Agonist	6.3245	6.536	C21 H27 N O5	-0.7	373.1887	80.39	9
Sakuranetin	Natural Product - Flavonoid	6.501	6.501	C16 H14 O5	-0.22	286.0841		
Styrene oxide	Industrial Chemical - Epoxide Intermediate	5.417	5.395	C8 H8 O	-0.89	120.0574		
Tucaresol	Pharmaceutical - Immunopotentiator	6.921	6.921	C15 H12 O5	-0.4	272.0684		
Uridine	Pharmaceutical - Nucleoside (Nutritional/Neurological)	2.532	3.07	C9 H12 N2 O6	0.3	244.0696		
5-Amino-6-cyclohexyl-4-hydroxy-2-isopropyl-hexanoic acid	Pharmaceutical - Experimental compound	5.83221	6.10345		80.39	272.2222		
Aureothin	Pharmaceutical - Antibacterial (Natural Product)	5.24212	5.51336	C22H23NO6	80.39	398.1594		
Distigmine	Pharmaceutical - Acetylcholinesterase Inhibitor	4.8424	5.11364	C22H32N4O4	80.39	415.2336	80.39	6
N,N-Dimethyldodecylamine-N-oxide	Pharmaceutical - Urea Derivative (Experimental)	5.98551	6.25675	C14H31NO	80.39	230.2478		
Phencyclidine	Pharmaceutical - Dissociative Anaesthetic	5.3147	5.58594	C17H25N	80.39	244.2061		
Phyto sphingosine	Pharmaceutical - Lipid (Skin Barrier Agent)	6.20293	6.47417	C18H39NO3	80.39	318.3		

2,3-Dihydroxybenzoic acid	Pharmaceutical - Iron chelator (natural)	4.461	4.515	C7H6O4	-0.2	154.0266		
2,3-Dimethoxy-5,6-dimethyl-p-benzoquinone	Industrial chemical - Quinone derivative	5.09	5.527	C10H12O4	-0.36	196.0736		
2,5,8,11,14-Pentaoxapentadecane	Industrial chemical - Polyether	3.092	3.092	C10H22O5	0.1	222.1467		
2-Phenyl-1H-imidazole-4-carboxylic acid	Pharmaceutical - Antifungal (imidazole)	5.045	5.09	C10H10N2O3	-0.38	206.0691		
3-(3,4-Dimethoxyphenyl)propionic acid	Pharmaceutical - Natural product analog	5.244	5.381	C11H14O4	-0.8	210.0892		
4-Aminobenzoic acid	Pharmaceutical - Vitamin B complex	4.491	4.985	C7H7NO2	-0.24	137.0477		
Acetovanillone	Flavourant	5.485	5.485	C9H10O3	-0.87	166.063		
Apigenin	Pharmaceutical - Flavonoid	7.941	7.941	C15H10O5	0.41	270.0528		
Aspirin	Pharmaceutical - NSAID	5.168	5.008	C9H8O4	-0.26	180.0423		
Benzoic acid	Pharmaceutical - Preservative / Industrial Chemical	5.1405	5.401	C7H6O2	-0.18	122.0368		
Ethofumesate	Agri Chem - Herbicide	4.6355	4.744	C13H18O5S	0.68	286.0875		
Gemcabene	Pharmaceutical - Lipid-lowering Agent	6.75	6.868	C16H30O5	0.52	302.2093		
Hydrocinnamic acid	Industrial Chemical - Flavoring Agent	5.413	5.413	C9H10O2	-0.12	150.0681		
Isovaleric acid	Industrial Chemical - Fragrance Ingredient	4.6095	4.558	C5H10O2	0.16	102.0681		
Nanafrocin	Pharmaceutical - Antifungal (Polyene-like)	6.9905	7.082	C16H14O6	0.57	302.079		
S-2-Hydroxyoctanoic acid	Pharmaceutical - Metabolite (Fatty Acid Derivative)	5.764	5.752	C8H16O3	-0.3	160.1099		
Styrene oxide	Industrial Chemical - Epoxide Intermediate	5.417	5.395	C8H8O	-0.88	120.0575		
Tert-Butylhydroquinone	Industrial Chemical - Food Preservative	5.418	5.712	C10H14O2	-0.66	166.0994		
Tucaresol	Pharmaceutical - Immunopotentiator	6.921	6.921	C15H12O5	-0.4	272.0685	78.33	20

Xanthine	Pharmaceutical - Purine Derivative (Metabolite)	2.3665	2.098	C5H4N4O2	-0.12	152.0334		
12-Hydroxydodecanoic acid	Industrial chemical - Fatty acid derivative	8.623	7.85596	C12H24O3	78.33	215.1652		
2,3-Dihydroxybenzoic acid	Pharmaceutical - Iron chelator (natural)	4.461	4.68865	C7H6O4	78.33	153.0193		
2-Phenyl-1H-imidazole-4-carboxylic acid	Pharmaceutical - Antifungal (imidazole)	5.045	4.7483	C10H10N2O3	78.33	169.0407		
3-(3,4-Dimethoxyphenyl)propionic acid	Pharmaceutical - Natural product analogue	5.244	5.0939	C11H14O4	78.33	209.0818		
4-Aminobenzoic acid	Pharmaceutical - Vitamin B complex	4.491	5.50818	C7H7NO2	78.33	136.0404		
Acetovanillone	Personal care product /everyday use- Fragrant or Flavouring agent	5.485	5.08154	C9H10O3	78.33	165.0555		
Apigenin	Pharmaceutical - Flavonoid	7.941	8.17479	C15H10O5	78.33	269.0457		
Aspirin	Pharmaceutical - NSAID	5.168	5.61303	C9H8O4	78.33	179.0349		
Benzoic acid	Pharmaceutical - Preservative / Industrial Chemical	5.1405	5.11364	C7H6O2	78.33	121.0295		
Hydro cinnamic acid	Industrial Chemical - Flavouring Agent	5.413	5.88281	C9H10O2	78.33	149.0607		
Isovaleric acid	Industrial Chemical - Fragrance Ingredient	4.6095	4.68865	C5H10O2	78.33	249.1344		
Phenylacetic acid	Naturally occurring (in honey)	4.987	4.7483	C8H8O2	78.33	135.0451		
S-2-Hydroxyoctanoic acid	Pharmaceutical - Metabolite (Fatty Acid Derivative)	5.764	5.51336	C8H16O3	78.33	159.1027		
Styrene oxide	Industrial Chemical - Epoxide Intermediate	5.417	5.58594	C8H8O	78.33	165.0557	78.33	14
(9Z,12R)-12-Hydroxyoctadec-9-enoic acid	Industrial chemical – surfactant/emulsifier	7.83403	8.10527	C18H34O3	75.72	298.2509	75.52	3
N,N-Dimethyldodecylamine-N-oxide	Pharmaceutical - Urea Derivative (Experimental)	5.98551	6.25675	C14H31NO	75.72	229.2404		

Phyto sphingosine	Pharmaceutical - Lipid (Skin Barrier Agent)	6.20293	6.47417	C18H39NO3	75.72	317.2927		
4-(2-Aminoethoxy)-n-(2,5-diethoxyphenyl)-3,5-dimethylbenzamide	Pharmaceutical - Experimental ligand	5.24212	5.51336		75.49	372.2045		
Albuterol	Pharmaceutical - Beta2 agonist	5.48923	5.76047	C13H21NO3	75.49	239.1521		
N,N-Dimethyldodecylamine-N-oxide	Pharmaceutical - Urea Derivative (Experimental)	5.98551	6.25675	C14H31NO	75.49	229.2404	75.49	5
Phytosphingosine	Pharmaceutical - Lipid (Skin Barrier Agent)	6.20293	6.47417	C18H39NO3	75.49	317.2927		
Yohimbine	Pharmaceutical - Alkaloid (Stimulant)	4.6898	4.96104	C21H26N2O3	75.49	354.1941		
5-Amino-6-cyclohexyl-4-hydroxy-2-isopropyl-hexanoic acid	Pharmaceutical - Experimental compound	5.83221	6.10345		75.15	271.2149		
N,N-Dimethyldodecylamine-N-oxide	Pharmaceutical - Urea Derivative (Experimental)	5.98551	6.25675	C14H31NO	75.15	229.2404		
Phyto sphingosine	Pharmaceutical - Lipid (Skin Barrier Agent)	6.20293	6.47417	C18H39NO3	75.15	317.2927	75.15	3
5-Amino-6-cyclohexyl-4-hydroxy-2-isopropyl-hexanoic acid	Pharmaceutical - Experimental compound	5.83221	6.10345		75.13	271.2149		
Albuterol	Pharmaceutical - Beta2 agonist	5.48923	5.76047	C13H21NO3	75.13	239.1521		
Aureothin	Pharmaceutical - Antibacterial (Natural Product)	5.24212	5.51336	C22H23NO6	75.13	397.1523		
Cyclohexyl-norstatine	Pharmaceutical - Protease Inhibitor	5.94417	6.21541		75.13	243.1834	75.13	8
Metoprolol	Pharmaceutical - Beta-blocker	7.76597	8.03721	C15H25NO3	75.13	267.1836		
N,N-Dimethyldodecylamine-N-oxide	Pharmaceutical - Urea Derivative (Experimental)	5.98551	6.25675	C14H31NO	75.13	229.2404		
N-(1-Adamantyl)-N'-(4-Guanidinobenzyl)urea	Pharmaceutical - Urea Derivative	5.24212	5.51336		75.13	341.2201		

	(Experimental)							
Phyto sphingosine	Pharmaceutical - Lipid (Skin Barrier Agent)	6.20293	6.47417	C18H39NO3	75.13	317.2927		
5-Amino-6-cyclohexyl-4-hydroxy-2-isopropyl-hexanoic acid	Pharmaceutical - Experimental compound	6.19476	6.10345		74.37	271.2149		
Ethyl morphine	Pharmaceutical - Opioid Analgesic	5.24368	5.51492	C19H23NO3	74.37	313.1673	74.37	4
N,N-Dimethyldodecylamine-N-oxide	Pharmaceutical - Urea Derivative (Experimental)	5.98551	6.25675	C14H31NO	74.37	229.2404		
Phyto sphingosine	Pharmaceutical - Lipid (Skin Barrier Agent)	6.20293	6.47417	C18H39NO3	74.37	317.2927		
1-O-Hexyl-2,3,5-trimethylhydroquinone	Industrial chemical - Antioxidant	5.45948	5.73072	C15H24O2	73.94	236.1776		
5-Amino-6-cyclohexyl-4-hydroxy-2-isopropyl-hexanoic acid	Pharmaceutical - Experimental compound	5.83221	6.10345		73.94	271.2149		
Phyto sphingosine	Pharmaceutical - Lipid (Skin Barrier Agent)	6.59576	6.47417	C18H39NO3	73.94	317.2927	73.94	3
Benzoic acid, 4-hydroxy-3,5-dimethoxy-, methyl ester	Pharmaceutical - Preservative / Industrial Chemical	5.61157	5.88281	C10H12O5	73.89	212.0684		
N,N-Dimethyldodecylamine-N-oxide	Pharmaceutical - Urea Derivative (Experimental)	6.598	6.25675	C14H31NO	73.89	229.2404		3
Phyto sphingosine	Pharmaceutical - Lipid (Skin Barrier Agent)	6.20293	6.47417	C18H39NO3	73.89	317.2927	73.89	

SI Table 3: CECs identified in a range of beehives at a higher confidence interval levels 3-1.

% of arable land <2km of hives	Name	Class	Formula	MW	m/z	RT	Pred RET	Confidence
84.94	1-Tetradecylamine	Urban agent	C14 H31 N	213.24563	214.2529	7.561	NA/needed	2a
84.94	Bis(2-ethylhexyl) amine	Urban agent	C16 H35 N	241.27702	242.28429	7.944	NA/needed	2a
84.94	Dodecyltrimethylammonium	Surfactant	C15 H33 N	227.26108	228.26835	7.587	NA/needed	2a
84.94	D-Sphingosine	Other	C18 H37 N O2	299.28252	300.28979	8.213	NA/needed	2a
84.94	Acetovanillone	Personal care product - fragrant	C9 H10 O3	166.06281	165.05551	5.485	6.16	2a
84.94	Aspirin	Pharmaceutical - NSAID	C9 H8 O4	180.04221	179.03494	5.008	4.91	2b
84.94	Phenylacetic acid	Naturally occuring (honey)	C8 H8 O2	136.0524	135.04512	5.557	5.59	3
84.94	1-Tetradecylamine	Urban agent	C14H31N	213.24564	214.2529	7.561	NA/needed	3
84.94	Thionazin	Agri chem-	C8 H13 N2 O3	248.038	249.045	12.9		2b

		Pesticide	P S	5	57	2		
89.63	Azoxystrobin	Agri chem - Fungicide	C22 H17 N3 O5	403.116 22	426.105 37	8.36 8	NA/need ed	2a
89.63	Sodium [dodecanoyl(methyl)amino]acetate	Industrial - Surfactant	C15 H29 N O3	271.214 99	272.222 27	9.06 5	NA/need ed	2a
89.63	4-Hydroxypropranolol	Pharmaceutical - Beta-blocker	C16 H21 N O3	275.152 18	276.159 46	9.27	NA/need ed	2a
89.63	Octyl hydrogen phthalate	Plasticizer	C16 H22 O4	278.152 03	279.159 32	0.63 9	NA/need ed	2a
89.63	Ibuprofen	Pharmaceutical - NSAID	C13 H18 O2	206.130 5	207.137 7	6.61 2	NA/need ed	2a
89.63	Thionazin	Agri chem- Pesticide	C8 H13 N2 O3 P S	248.038 5	249.045 57	12.9 2		2b
89.63	Stearamide	Personal care product	C18 H37 N O	283.287 6	284.294 88	10.9 61	NA/need ed	2a
90.98	Dodecyltrimethylammonium	Surfactant	C15 H33 N	227.261 08	228.268 35	7.58 7	NA/need ed	3
90.98	4-Hydroxypropranolol	Pharmaceutical - Beta-blocker	C16 H21 N O3	275.152 18	276.159 46	9.27	NA/need ed	2a
90.98	Stearamide	Personal care product	C18 H37 N O	283.287 6	284.294 88	10.9 61	NA/need ed	2a
74.37	N-Butylbenzenesulfonamide	Plasticizer	C10 H15 N O2 S	213.082 37	214.089 64	0.64 8	NA/need ed	2a
74.37	N,N-Dimethyldodecylamine-N-oxide	Surfactant/Antimicro bial	C14 H31 N O	229.240 41	230.247 7	6.86 8	NA/need ed	2a
74.37	Alprenolol	Pharmaceutical - Beta-blocker	C15 H23 N O2	249.172 9	250.180 19	8.88 6	NA/need ed	2a
74.37	Amitriptyline	Pharmaceutical - Antidepressant	C20 H23 N	277.183 16	278.190 44	6.38	NA/need ed	2a
74.37	Carbamazepine	Pharmaceutical - Anticonvulsant	C15 H12 N2 O	236.094 86	237.102 07	6.36 4	NA/need ed	2a
74.37	N-Butylbenzenesulfonamide	Plasticizer	C10 H15 N O2 S	213.082 37	214.089 64	0.64 8	NA/need ed	2a
74.37	Viloxazine	Pharmaceutical - Antidepressant	C13 H19 N O3	237.136 6	238.143 8	5.58	NA/need ed	2a
74.37	Warfarin Alcohol	Pharmaceutical - anticoagulant	C19 H18 O4	310.120 7	311.127 9	5.14 8	NA/need ed	2b
74.37	Combretastatin	Pharmaceutical –	C18 H22 O6	334.141	357.130	5.62	5.62822	3

		Vascular Disrupting Agent		59	79	9	5	
74.37	Thionazin	Agri chem-Pesticide	C8 H13 N2 O3 P S	248.0385	249.04557	12.92		2b
83.93	Estriol	Hormone	C18 H24 O3	288.17237	311.1615	7.239	NA/needed	2a
83.93	(1R,2S,3S,5S,11R,12R)-5-(furan-3-yl)-12-hydroxy-3,11-dimethyl-6,14-dioxatetracyclo[10.2.2.0Â?,ÂaÂa.0ÂI,â¸]hexadec-15-ene-7,13-dione	Other	C20 H22 O6	358.14127	359.14867	7.009	NA/needed	2a
83.93	4-{5-[(3S)-1-(3,4-Difluorobenzyl)-3-pyrrolidinyl]-1,3,4-oxadiazol-2-yl}-N,N-dimethylaniline	Metabolite/other	C21 H22 F2 N4 O	384.17575	385.183	6.517	NA/needed	2a
83.93	4-Hydroxypropranolol	Pharmaceutical - Beta-blocker	C16 H21 N O3	275.15218	276.15946	9.27	NA/needed	2a
83.93	2,3-Dihydroxybenzoic acid	Pharmaceutical - Antioxidant	C7 H6 O4	154.02658	153.0193	4.515	4.48	2b
83.93	Acetovanillone	Personal care product - fragrant	C9 H10 O3	166.06281	165.05551	5.485	6.16	2b
83.93	Isovaleric acid	Pharmaceutical - Organic Compound	C5 H10 O2	102.0681	249.13441	4.558	4.6	2b
83.93	Phenylacetic acid	Naturally occurring (honey)	C8 H8 O2	136.0524	135.04512	5.557	5.59	3
83.93	Thionazin	Agri chem-Pesticide	C8 H13 N2 O3 P S	248.0385	249.04557	12.92		2b
75.13	Albuterol	Pharmaceutical - Bronchodilator	C13 H21 N O3	239.15206	240.1593	5.068	NA/needed	2a
75.13	Aureothin	Pharmaceutical - Antibiotic	C22 H23 N O6	397.15233	398.1596	5.93	NA/needed	2a
75.13	Cyclohexyl-norstatine	Pharmaceutical - Anti-inflammatory	C13 H25 N O3	243.18342	244.1907	6.401	NA/needed	2a
75.13	Metoprolol	Pharmaceutical - Beta-blocker	C15 H25 N O3	267.1836	268.1909	7.699	NA/needed	2a
75.13	N,N-Dimethyldodecylamine-N-oxide	Surfactant	C14 H31 N O	229.24041	230.2477	6.868	NA/needed	2a
75.13	Nortriptyline	Pharmaceutical - Antidepressant	C19 H21 N	263.16754	264.17481	6.036	NA/needed	2a
75.13	Nuarimol	Agri Chem - Fungicide	C17 H12 Cl F N2 O	314.06136	315.06883	1.611	NA/needed	2a

75.13	Pirbuterol	Pharmaceutical - Bronchodilator	C12 H20 N2 O3	240.1474	241.1547	2.853	NA/needed	2a
75.13	Sodium [dodecanoyl(methyl)amino]acetate	Pharmaceutical/Surfactant	C15 H29 N O3	271.21499	272.22227	9.065	NA/needed	2a
75.13	Cycloheximide	Pharmaceutical - antibiotic	C15 H23 N O4	281.1627	282.16998	5.826	NA/needed	2b
80.39	Cyclophosphamide	Pharmaceutical - Anticancer	C7 H15 Cl2 N2 O2 P	260.02484	261.03211	5.557	NA/needed	2a
80.39	Alprenolol	Pharmaceutical - Beta-blocker	C15 H23 N O2	249.1729	250.18019	8.886	NA/needed	2a
80.39	Dodecyl trimethylammonium	Industrial-Domestic/Disinfectant	C15 H33 N	227.26108	228.26835	7.587	NA/needed	2a
80.39	Flurandrenolide	Pharmaceutical - Corticosteroid	C24 H33 F O6	436.2278	437.23508	4.107	NA/needed	2a
80.39	Aureothin	Pharmaceutical - Antibiotic	C22 H23 N O6	397.15233	398.1596	5.93	NA/needed	2a
80.39	Distigmine	Pharmaceutical - Cholinesterase inhibitor	C22 H32 N4 O4	416.24053	439.2297	5.341	NA/needed	2a
80.39	N,N-Dimethyldodecylamine-N-oxide	Surfactant	C14 H31 N O	229.24041	230.2477	6.868	NA/needed	2a
80.39	Carbamazepine	Pharmaceutical - Anticonvulsant	C15 H12 N2 O	236.09486	237.10207	6.364	NA/needed	2a
80.39	Cyclophosphamide	Pharmaceutical - Anticancer	C7 H15 Cl2 N2 O2 P	260.02484	261.03211	5.557	NA/needed	2a
80.39	Sodium [dodecanoyl(methyl)amino]acetate	Pharmaceutical - Surfactant	C15 H29 N O3	271.21499	272.22227	9.065	NA/needed	2a
80.39	Ritobegron	Pharmaceutical - Beta-3 Agonist	C21 H27 N O5	373.18866	372.18138	6.536	6.33	3
80.39	Styrene oxide	Industrial Chemical	C8 H8 O	120.05746	165.05566	5.395	5.43	2b
80.39	Thionazin	Agri chem-Pesticide	C8 H13 N2 O3 P S	248.0385	249.04557	12.92		2b
80.39	4-Aminobenzoic acid	Pharmaceutical/personal care product Preservative (metabolite)	C7 H7 N O2	137.04765	136.04037	4.985	4.49	3

75.72	Trimethoprim	Pharmaceutical - Antibiotic	C14 H18 N4 O3	290.13751	291.14478	4.067	NA/needed	2a
75.72	N,N-Dimethyldodecylamine-N-oxide	Surfactant	C14 H31 N O	229.24041	230.2477	6.868	NA/needed	2a
75.72	Alminoprofen	Pharmaceutical - NSAID	C13 H17 N O2	219.12589	220.13317	6.722	NA/needed	3
75.72	Nortriptyline	Pharmaceutical - Antidepressant	C19 H21 N	263.16754	264.17481	6.036	NA/needed	2a
75.72	Toliprolol	Over the counter drug	C13 H21 N O2	201.17289	224.16209	9.043	NA/needed	2a
75.72	Warfarin Alcohol	Pharmaceutical - anticoagulant	C19 H18 O4	310.1207	311.1279	5.148	NA/needed	2b
73.94	Carbamazepine	Pharmaceutical - Anticonvulsant	C15 H12 N2 O	236.09486	237.10207	6.364	NA/needed	2a
73.94	Combretastatin	Pharmaceutical – Vascular Disrupting Agent	C18 H22 O6	334.14159	357.13079	5.629	5.628225	3
73.94	Sulfadiazine	Pharmaceutical - antibiotic	C10 H10 N4 O2 S	250.05263	251.0599	1.296	NA/needed	2b
81.12	4-Hydroxypropranolol	Pharmaceutical- Beta-blocker	C16 H21 N O3	275.15218	276.15946	9.27	NA/needed	2a
81.12	Alprenolol	Pharmaceutical - Beta-blocker	C15 H23 N O2	249.1729	250.18019	8.886	NA/needed	2a
81.12	Acetovanillone	Personal care product - fragrant	C9 H10 O3	166.06281	165.05551	5.485	6.16	2b
81.12	Aspirin	Pharmaceutical - NSAID	C9 H8 O4	180.04221	179.03494	5.008	4.91	2b
81.12	Styrene oxide	Industrial Chemical - Epoxide	C8 H8 O	120.05746	165.05566	5.395	5.43	2b
81.12	Aspirin	Pharmaceutical - NSAID	C9 H8 O4	180.04221	179.03494	5.008	4.91	2b
81.12	Squaric acid dibutyl ester	Pharmaceutical - Topical Agent	C12 H18 O4	226.12045	225.11316	5.43	6.82	2b
81.12	Styrene oxide	Industrial Chemical - Epoxide	C8 H8 O	120.05746	165.05566	5.395	5.43	2b
81.12	Phenylacetic acid	Naturally occurring (honey)	C8 H8 O2	136.0524	135.04512	5.557	5.59	3
81.12	Thionazin	Agri chem- Pesticide	C8 H13 N2 O3 P S	248.0385	249.04557	12.92		2b

78.33	2,3-Dimethoxy-5,6-dimethyl-p-benzoquinone	Research chemical	C10 H12 O4	196.07349	195.06621	5.527	4.91	2b
78.33	Aspirin	Pharmaceutical - NSAID	C9 H8 O4	180.04221	179.03494	5.008	4.91	2b
78.33	Styrene oxide	Industrial Chemical	C8 H8 O	120.05746	165.05566	5.395	5.43	2b
78.33	Thionazin	Agri chem-Pesticide	C8 H13 N2 O3 P S	248.0385	249.04557	12.92		2b
78.33	4-Aminobenzoic acid	Preservative (metabolite)	C7 H7 N O2	137.04765	136.04037	4.985	4.49	3
78.33	Thionazin	Agri chem-Pesticide	C8 H13 N2 O3 P S	248.0385	249.04557	12.92		2b
75.15	N-Butylbenzenesulfonamide	Plasticizer	C10 H15 N O2 S	213.08237	214.08964	0.648	NA/needed	2a
75.15	5-Amino-6-cyclohexyl-4-hydroxy-2-isopropyl-hexanoic acid	Research chemical	C15 H29 N O3	271.2149	272.2222	6.778	NA/needed	2a
75.15	Combretastatin	Pharmaceutical – Vascular Disrupting Agent	C18 H22 O6	334.14188	333.13461	6.18	6.17	3
75.15	N,N-Dimethyldodecylamine-N-oxide	Surfactant/Antimicrobial	C14 H31 N O	229.24041	230.2477	6.868	NA/needed	2a
75.15	(3R,4S)-6,8-Dihydroxy-3,4,5-trimethyl-1-oxo-3,4-dihydro-1H-isochromene-7-carboxylic acid	Pharmaceutical - Anti-inflammatory	C13 H14 O6	266.07916	267.08644	4.09	NA/needed	2a
75.15	Ajmaline	Pharmaceutical - Antiarrhythmic	C20 H26 N2 O2	326.19959	327.20687	5.427	NA/needed	2a
75.15	Danazol	Pharmaceutical - Androgenic Steroid	C22 H27 N O2	337.20417	338.21145	5.967	NA/needed	2a
75.15	N-Butylbenzenesulfonamide	Plasticizer	C10 H15 N O2 S	213.08237	214.08964	0.648	NA/needed	2a
75.15	Nuarimol	Agri Chem - Fungicide	C17 H12 Cl F N2 O	314.06136	315.06883	1.611	NA/needed	2a
75.15	Oxitropium	Pharmaceutical - Anticholinergic	C19 H25 N O4	331.17829	332.18557	4.408	NA/needed	2a
75.15	Tetramethrin	Agri Chem - Insecticide	C19 H25 N O4	331.17843	332.18571	4.966	NA/needed	2a
73.89	N,N-Dimethyldodecylamine-N-oxide	Surfactant/Antimicrobial	C14 H31 N O	229.24041	230.2477	6.868	NA/needed	2a

73.89	Flurandrenolide	Pharmaceutical - Corticosteroid	C24 H33 F O6	436.227 8	437.235 08	4.10 7	NA/need ed	2a
73.89	Nuarimol	Agri Chem - Fungicide	C17 H12 Cl F N2 O	314.061 36	315.068 83	1.61 1	NA/need ed	2a
73.89	Combretastatin	Pharmaceutical – Vascular Disrupting Agent	C18 H22 O6	334.141 59	357.130 79	5.62 9	5.62822 5	3
86.13	Dimethyl sebacate	Plasticizer	C12 H22 O4	230.151 64	231.158 89	7.84	NA/need ed	2a
86.13	Trinexapac	Plant growth regulator	C11 H12 O5	224.068 6	225.075 9	3.99 3	NA/need ed	3
86.13	4-{6-[2-(4-carboxyphenyl)-1H-benzo[d]imidazol-5-yl]-1H-benzo[d]imidazol-2-yl}benzoic acid	Pharmaceutical - Metabolite	C28 H18 N4 O4	474.134 47	475.141 67	3.76 9	NA/need ed	3
86.13	Aspirin	Pharmaceutical - NSAID	C9 H8 O4	180.042 21	179.034 94	5.00 8	4.91	2b
86.13	Methyldopa	Pharmaceutical - Antihypertensive	C10 H13 N O4	211.084 31	212.091 6	6.43 4	3.55	2b
86.13	Phenylacetic acid	Naturally occurring (honey)	C8 H8 O2	136.052 4	135.045 12	5.55 7	5.59	3
86.13	Styrene oxide	Industrial Chemical	C8 H8 O	120.057 46	165.055 66	5.39 5	5.43	2b
86.13	Thionazin	Agri chem- Pesticide	C8 H13 N2 O3 P S	248.038 5	249.045 57	12.9 2		2b
75.49	Albuterol	Pharmaceutical - Bronchodilator	C13 H21 N O3	239.152 06	240.159 3	5.06 8	NA/need ed	2a
75.49	N,N-Dimethyldodecylamine-N-oxide	Surfactant	C14 H31 N O	229.240 41	230.247 7	6.86 8	NA/need ed	2a
75.49	Nortriptyline	Pharmaceutical - Antidepressant	C19 H21 N	263.167 54	264.174 81	6.03 6	NA/need ed	2a
75.49	Cycloheximide	Agri-chem Fungicide	C15 H23 N O4	281.162 7	282.169 98	5.82 6	NA/need ed	2b
73.96	N-Butylbenzenesulfonamide	Plasticizer	C10 H15 N O2 S	213.082 37	214.089 64	0.64 8	NA/need ed	2a
73.96	N,N-Dimethyldodecylamine-N-oxide	Surfactant	C14 H31 N O	229.240 41	230.247 7	6.86 8	NA/need ed	2a
73.96	Alminoprofen	Pharmaceutical - NSAID	C13 H17 N O2	219.125 89	220.133 17	6.72 2	NA/need ed	2b

73.96	N-Butylbenzenesulfonamide	Plasticizer	C10 H15 N O2 S	213.08237	214.08964	0.648	NA/needed	2a
73.96	Tiaprofenic acid	Pharmaceutical - NSAID	C14 H12 O3 S	260.05096	261.05824	1.081	NA/needed	2a
73.96	Warfarin Alcohol	Pharmaceutical - anticoagulant	C19 H18 O4	310.1207	311.1279	5.148	NA/needed	2b
73.96	Combretastatin	Pharmaceutical – Vascular Disrupting Agent	C18 H22 O6	334.14159	357.13079	5.629	5.628225	3

Footnote – *NA/needed indicates identification was achieved using the automated Compound Discoverer otherwise RT predictions were used.*

SI Table 4: Contaminants present in shop bought honey (control) - Level 3-2a.

**Chemical**	**Peak area**	**Peak rating**	**Database**
(2E)-3-(3,4-dimethoxyphenyl)prop-2-enoic acid	330283.4	1.4	Level 2a
(2E,4E)-N-(1,3-dihydroxy-3-methylpentan-2-yl)-2-methylhexa-2,4-dienamide	4715022	5.3	Level 2a
(2E,4E,9Z)-1-(piperidin-1-yl)hexadeca-2,4,9-trien-1-one	33222764	3.5	Level 2a
(2R,3R,4S,5S,6R)-2-{[(2E,6R)-6-hydroxy-2,6-dimethylocta-2,7-dien-1-yl]oxy}-6-(hydroxymethyl)oxane-3,4,5-triol	3942015	7	Level 2a
(2S,5aS,8aR)-2-{3-[(2R)-2-(Methoxymethyl)-1-pyrrolidinyl]-3-oxopropyl}-1,6-dimethyloctahydropyrrolo[3,2-E][1,4]diazepin-5(2H)-one	2232415	8.3	Level 2a

(3aR,4aS,5R,7aS,8S,9aR)-5-Hydroxy-4a,8-dimethyl-3-methyleneoctahydroazuleno[6,5-b]furan-2,6(3H,4H)-dione	718904.4	7.4	Level 2a
(3aR,7aS,8S,9aR)-5,8-dimethyl-3-methylidene-2H,3H,3aH,4H,6H,7H,7aH,8H,9H,9aH-azuleno[6,5-b]furan-2,6-dione	76625976	7.8	Level 2a
Abscisic acid	27912332	9.5	Level 2a
1-(3-acetyl-2,4,6-trihydroxyphenyl)ethan-1-one	35590549	7.8	Level 2a
1-(3-chlorophenyl)-4-{2-[1-(4-chlorophenyl)-1H-1,2,3,4-tetraazol-5-yl]vinyl}piperazine	1276744	8.3	Level 2a
1-[4-hydroxy-3-(3-methylbut-2-en-1-yl)phenyl]ethan-1-one	2769906	4.8	Level 2a
1-{[4-Hydroxy-6-(methoxymethyl)pyrimidin-2-yl]thio}-3,3-dimethylbutan-2-one	4905447	8	Level 2a
2-Amino-1,3,4-octadecanetriol	51065240	9.5	Level 2a
2-Styryl-1,3-benzoxazole	21941879	9.5	Level 2a
3-[2,6-Dinitro-4-(trifluoromethyl)anilino]-2-azepanone	463657.6	6.8	Level 2a
3?butyl?1?{4?[(1E)?2?(4?nitrophenyl)diazen?1?yl]phenyl}urea	1401640	6.1	Level 2a
4,4'-Sulfonylbis[2-(prop-2-en-1-yl)phenol]	9722488	2.9	Level 2a
4,6,8-trihydroxy-7-methoxy-3-methyl-3,4-dihydro-1H-2-benzopyran-1-one	90740275	8.9	Level 2a
4-(2-chloro-6-fluorobenzyl)-3,5-dimethyl-4-prop-2-ynyl-4H-pyrazole	12732561	6.7	Level 2a
4-(4-chlorophenoxy)-3,5-dimethyl-1H-pyrazole	36243085	8.4	Level 2a
4-(4-hydroxy-3,5-dimethylphenyl)-2-methylphthalazin-1(2H)-one	872153.5	5.2	Level 2a
4-(Dimethylamino)benzophenone	4613512	9.1	Level 2a
4-(methylthio)-6-phenyl-2-(3-pyridyl)pyrimidine-5-carbonitrile	5187722	9.1	Level 2a

4-[(6E)-3-Hydroxy-8,10-dimethyl-2-(methylamino)-6-dodecen-1-yl]phenol	1278014	8.3	Level 2a
4-Methoxy-N,N-dimethylcathinone	14796961	9.5	Level 2a
4-Pyridinyl{(3S)-3-[5-(3-pyridinyl)-1,3,4-oxadiazol-2-yl]-1-pyrrolidinyl}methanone	1170740	8.8	Level 2a
4-{6-[2-(4-carboxyphenyl)-1H-benzo[d]imidazol-5-yl]-1H-benzo[d]imidazol-2-yl}benzoic acid	1286451	4.5	Level 2a
5-Chloro-2-[(3S)-1-(4-fluorobenzyl)-3-pyrrolidinyl]-1,3-benzoxazole	6547568	3.2	Level 2a
5-Methyl-N'-({[3-(trifluoromethyl)anilino]carbonyl}oxy)isoxazole-3-carboximidamide	1.44E+08	8.4	Level 2a
6-Hydroxy-8-methoxy-3-methyl-3,4-dihydro-1H-isochromen-1-one	409688.7	3	Level 2a
7-(2-aminophenyl)heptanoic acid	7642093	9.1	Level 2a
8-(1,2-dihydroxy-3-methylbut-3-en-1-yl)-7-methoxy-2H-chromen-2-one	100738.2	2.2	Level 2a
8-{[(3S)-3-(1H-Benzimidazol-2-yl)-1-pyrrolidinyl]sulfonyl}quinoline	4103703	5.3	Level 2a
AICA ribonucleotide	1896731	5.5	Level 2a
Alverine	105525.6	6.5	Level 2a
Arachidonic acid-biotin	824790.7	6.8	Level 2a
Argininosuccinic acid	14356528	10	Level 2a
Atrazine	26232670	10	Level 2a
Basic Violet 1	42299595	9.5	Level 2a
Bis(2-ethylhexyl) amine	362431.5	3	Level 2a

Clofilium	1060459	7.2	Level 2a
Decarbamoyl-neosaxitoxin	3129863	4.8	Level 2a
Diisooctyl phthalate	50883.38	7.1	Level 2a
Dimethachlor OXA	2671706	6.4	Level 2a
DNH	35449391	4	Level 2a
Docosanamide	4937770	3.2	Level 2a
Dodecyltrimethylammonium	1103675	6.6	Level 2a
DQH	3839186	4.2	Level 2a
Drospirenone	752246.9	3.6	Level 2a
Erucamide	8552958	3.7	Level 2a
Ethamivan	6875963	7	Level 2a
Gentian violet	55404996	7.8	Level 2a
INH	6052913	9.1	Level 2a
mesityl (4-methylphenyl) sulfone	5104292	9.1	Level 2a
N'5-(2-chlorobenzylidene)-4-methyl-2-(1H-pyrrol-1-yl)-1,3-thiazole-5-carbohydrazide	13453116	8.4	Level 2a
N,N-dimethyl-5-nitro-6-[3-(trifluoromethyl)phenoxy]pyrimidin-4-amine	22793068	1.8	Level 2a
N-(9-oxodecyl)acetamide	2263365	7.7	Level 2a
N-[3-(2-methyl-4-pyrimidinyl)phenyl]-1,3-benzothiazole-2-carboxamide	1.79E+08	9.5	Level 2a
N-{(1R,9S)-11-[(1-Methyl-1H-indol-3-yl)methyl]-6-oxo-7,11-diazatricyclo[7.3.1.02,7]trideca-2,4-dien-5-yl}benzamide	2649941	8.6	Level 2a
N1-(3-{[(2,2-dimethylhydrazino)carbonyl]amino}-4-methylphenyl)-2,2-dimethylhydrazine-1-carboxamide	452332.5	4.7	Level 2a
N1-(4-chlorophenyl)-2-({4-methyl-5-[1-methyl-2-(methylthio)-	6954992	4.2	Level 2a

1H-imidazol-5-yl]-4H-1,2,4-triazol-3-yl}thio)acetamide			
N1-cyclohexyl-2-[{2-[(4-chlorophenyl)thio]acetyl}(methyl)amino]benzamide	1326793	1.7	Level 2a
Nuarimol	451510.2	5.2	Level 2a
Octadecanamine	7848810	9.1	Level 2a
Palmitelaidic acid methyl ester	2941370	5.3	Level 2a
PEG n5	7598839	2.6	Level 2a
PPG n4	8777580	6.7	Level 2a
PPG n7	28192074	8.9	Level 2a
PPG n8	25996715	7.8	Level 2a
PQH	5480308	1.5	Level 2a
Prolintane	2751218	8	Level 2a
QKK	366695.7	2.5	Level 2a
Stearic acid	329927.9	7.4	Level 2a
TNH	24943538	4.6	Level 2a
TNH	3424821	6.4	Level 2a
Triphenylphosphine oxide	28704293	9.5	Level 2a
{3-Methyl-5-(5-methylisoxazol-3-yl)-1-[3-(trifluoromethyl)phenyl]-1H-pyrazol-4-yl}(morpholino)methanone	686708.3	8.5	Level 2a
(9Z,12R)-12-Hydroxyoctadec-9-enoic acid	207432.805	3.3	Level 3
(Z)-Hexadec-9-enoic acid	5536225.644	2.1	Level 3
(Z)-Hexadec-9-enoic acid	4044027.656	2.1	Level 3
1-Butanoyl-n-(4-carbamimidoylbenzyl)-l-prolinamide	4019986.412	4.2	Level 3
13-Formyl-12,14-dihydroxy-3,5,7-trimethyltetradeca-2,4-	1085986.322	6.1	Level 3

dienoic acid			
13-Hydroxyoctadeca-9,11-dienoic acid	301550.6973	4.9	Level 3
2,10,10-Trimethyltricyclo[6.3.0.0�?,?]undec-6-ene-6-carboxylic acid	14239763.91	7.3	Level 3
2-Butyl-1h,4h,5h,6h,7h-imidazo[4,5-d]pyridazine-4,7-dione	4839687.587	1.5	Level 3
3,4-Methylenedioxyethamphetamine	993313.9754	6.6	Level 3
3,6,9,12-Tetraoxaeicosan-1-ol	27722723.45	7.8	Level 3
5-{[2-(6-Amino-9H-purin-9-yl)ethyl]amino}pentan-1-ol	11039707.73	9.5	Level 3
Alpha-Amylcinnamaldehyde, (Z)-	7594597.534	7	Level 3
Alpha-bisabolol	25214554.94	5.7	Level 3
Antroquinonol	7690577.047	7	Level 3
Benserazide	1039140.297	3.9	Level 3
Bombykol	2230708.62	4.5	Level 3
Brd-a41145729-001-02-7	309732.6879	7.1	Level 3
Brd-a53416814-001-01-5	928748.1416	7.9	Level 3
Brd-a53416814-001-01-5	503298.5113	0.9	Level 3
Bufexamac	297207.0215	3.8	Level 3
Bunazosin	685749.9883	0.9	Level 3
Chir-265	18118617.98	4	Level 3
Chromanol	620428.5946	0.9	Level 3
Crotetamide	11778572.18	6.2	Level 3
Cyclohexyl-norstatine	2125404.1	6.6	Level 3

Decamethylcyclopentasiloxane	25392877.03	5.1	Level 3
Drospirenone	32317069.41	7.8	Level 3
Elaidoylamide	65975147.68	5.7	Level 3
Hexadecanal	386912.2685	0.9	Level 3
Hexadecanoic acid	30076886.75	8.4	Level 3
Isopropyl tetradecanoate	2723375.372	3.7	Level 3
N,N-Dimethyldodecylamine-N-oxide	5929368.866	7.5	Level 3
N-Butylbenzenesulfonamide	1974694.582	1.7	Level 3
Netilmicin	650496.4753	7.4	Level 3
Phenformin	2323197.552	7.2	Level 3
Phenol, 4-(butoxymethyl)-2-methoxy-	2231344.128	3.9	Level 3
Spiroxamine	1472018.423	3.9	Level 3
Stearic acid	21201635.46	8.4	Level 3
Tetradecanoic acid	492653.4323	1.4	Level 3
{[5-(2-Amino-6-hydroxy-9h-purin-9-yl)-3-hydroxyoxolan-2-yl]methoxy}({[5-(2-hydroxy-4-imino-1,4-dihydropyrimidin-1-yl)-2-(hydroxymethyl)oxolan-3-yl]oxy})phosphinic acid	16977867.68	5.7	Level 3

SI Table 5: Semi Quantified concentrations of pharmaceuticals present in UK beehives (honey) (Level 2-1).

Contaminant	Average concentration (ng/g dw)	Standard deviation	Frequency
Viloxazine	13	20	5
Ibuprofen	29	30	7
Carbamazepine	70	30	3
Cycloheximide	150	190	4
4-Hydroxypropranolol	14	3	6
Albuterol	5	4	6
Aspirin	360	380	7
Alprenolol	1	0.8	2
Estriol	8	5	5
Trimethoprim	20	3	3
Metoprolol	3	2	2
Flurandrenolide	580	340	5
Amitriptyline	0.6	0.3	1
Valomaciclovir	64	4	1
Sulfadiazine	30	30	2
Aureothin	1	0.6	4
Combretastatin	2	0.2	4
Warfarin alcohol	0.8	0.9	2
Tiaprofenic acid	0.5	0.3	2

SI References

1. Sigma-Aldrich. (n.d.) *Handheld Refractometer HMX-1, Honey Moisture, ATC* [product page]. Available at: https://www.sigmaaldrich.com/GB/en/product/sigma/z744765.

2. Schymanski, E. L., Jeon, J., Gulde, R., Fenner, K., Ruff, M., Singer, H. P., & Hollender, J. (2014). Identifying small molecules via high resolution mass spectrometry: communicating confidence. *Analytical and Bioanalytical Chemistry*.

3. NORMAN Network, 2024. NORMAN SusDat: NORMAN Substance Database. [online] Available at: https://www.norman-network.com/nds/susdat/ [Accessed 10 Oct. 2024].

4. ChemSpider, nd. ChemSpider: The free chemical structure database. [online] Available at: https://www.chemspider.com/

5. Stanstrup J, Neumann S, Vrhovšek U. PredRet: Prediction of Retention Time by Direct Mapping between Multiple Chromatographic Systems. Anal. Chem. 2015.

7. Aalizadeh, R., Nikolopoulou, V., Alygizakis, N., Slobodnik, J. and Thomaidis, N.S., (2022) 'A novel workflow for semi-quantification of emerging contaminants in environmental samples analyzed by LC-HRMS', Analytical and Bioanalytical Chemistry, 414, pp. 7435–7450.

8. Oss, M., Kruve, A., Herodes, K. and Leito, I., (2010) 'Electrospray ionization efficiency scale of organic compounds', Analytical Chemistry, 82, pp. 2865–2872.

9. Liigand, J., Kruve, A., Leito, I., Girod, M. and Antoine, R., 2014. Effect of mobile phase on electrospray ionization efficiency. Journal of The American Society for Mass Spectrometry, 25(11), pp.1853-1861.

10. Liigand, J., Wang, T., Kellogg, J., Smedsgaard, J., Cech, N. and Kruve, A., (2020) 'Quantification for non-targeted LC/MS screening without standard substances', Scientific Reports, 10, p. 5808.
